# Service coverage for major depressive disorder: estimated rates of minimally adequate treatment for 204 countries and territories in 2021

**DOI:** 10.1016/S2215-0366(24)00317-1

**Published:** 2024-12

**Authors:** Damian F Santomauro, Theo Vos, Harvey A Whiteford, Dan Chisholm, Shekhar Saxena, Alize J Ferrari

**Affiliations:** a Queensland Centre for Mental Health Research, Wacol, QLD, Australia; b The University of Queensland, School of Public Health, Herston, QLD, Australia; c Institute for Health Metrics and Evaluation, University of Washington, Seattle, WA, USA; d Department of Mental Health and Substance Use, World Health Organization, Geneva, Switzerland; e Harvard T H Chan, School of Public Health, Harvard University, Boston, MA, USA

## Abstract

**Background:**

Access to effective treatment for major depressive disorder remains limited and difficult to track across place and time. We analysed the available data on minimally adequate treatment (MAT) for major depressive disorder globally with the aim of providing a useful metric against which to monitor national responses to the growing public health burden imposed by major depressive disorder.

**Methods:**

MAT was defined as pharmacotherapy (1 month of medication, plus four visits to a medical doctor) or psychotherapy (eight visits with any professional). From existing reviews, we identified mental health surveys that assessed major depressive disorder within the general population as well as health service uptake by individuals with major depressive disorder. Data by ethnicity were not available. Estimates of MAT, antidepressant use, or use of any mental health service were extracted. The latter two estimates were adjusted to reflect likely MAT rates via a network meta-analysis. Adjusted MAT estimates were analysed via a Bayesian meta-regression using the Disease Modelling Meta-Regression (DisMod-MR 2.1) tool. This analysis estimated MAT coverage among people with major depressive disorder by age, sex, location, and year. Final MAT estimates were standardised by age and sex against the existing age and sex distribution of people with major depressive disorder globally. People with lived experience were involved in the design, preparation, interpretation, and writing of this manuscript.

**Findings:**

The analysed dataset included 145 estimates from 32 studies, covering 31 countries, 14 regions, and six super-regions. The proportion of people with major depressive disorder receiving MAT globally in 2021 was 9·1% (95% uncertainty interval 7·2–11·6), with 10·2% (8·2–13·1) of females and 7·2% (5·7–9·3) of males with major depressive disorder receiving MAT. MAT coverage was highest in high-income locations (27·0% [21·7–34·4]), with Australasia having the highest rate (29·2% [21·4–40·8]). MAT coverage was lowest in sub-Saharan Africa (2·0% [1·5–2·6]), within which western sub-Saharan Africa (1·8% [1·4–2·5]) had the lowest coverage. Seven countries (Australia, Belgium, Canada, Germany, the Netherlands, South Korea, and Sweden) were estimated to have MAT coverage exceeding 30%, while 90 countries were estimated to have coverage lower than 5%.

**Interpretation:**

Despite many gaps in the available data, estimates show that, globally, most individuals with major depressive disorder do not receive MAT. Services must improve to reach a global coverage that better meets the mental health needs of those with major depressive disorder. Urgent attention should be given to the scale-up of effective intervention strategies, especially in low-income and middle-income countries, as well as further research into better quality treatment options for major depressive disorder. We present a means by which the MAT gap for major depressive disorder can be quantified, to monitor and inform action by governments and international partners.

**Funding:**

Queensland Health and the Bill & Melinda Gates Foundation.

## Introduction

Major depressive disorder is a common and disabling mental disorder.^1^ It is characterised by frequent and persistent depressed mood or loss of pleasure or interest in daily activities that substantially affects daily functioning. Major depressive disorder places individuals at an increased risk of other diseases and injuries and early mortality (including suicide) compared with the general population. It also leads to many social, occupational, and economic consequences for individuals, their care-givers, and their broader community.^2^

Since their introduction in the 1990s, the Global Burden of Diseases, Injuries, and Risk Factors (GBD) Studies have emphasised that a large proportion of global burden or health loss is associated with mental disorders, and particularly major depressive disorder. GBD 2021, the latest GBD study, estimated 229 million (uncertainty interval [UI] 200–269) cases of major depressive disorder.^3^ The GBD ranking of health loss or disease burden by disease and injury placed major depressive disorder as the second leading level-4 cause of disability (as measured by years lived with disability) and the 15th leading cause of total burden (as measured by disability-adjusted life-years [DALYs]) globally in 2021.^3^ GBD estimates showed minimal global reduction in the burden from major depressive disorder between 1990 and 2019 (1·7% [UI 0·4–3·0] reduction in age-standardised DALYs), and then an increase of 17·6% (15·3–20·1) in 2020 and 18·1% (15·9–20·5) in 2021 since 2019 with the impact of the COVID-19 pandemic.^3^, ^4^

**Research in context**

**Evidence before this study**

We searched PubMed from database inception to Sept 16, 2024, for papers providing estimates of minimally adequate treatment (MAT) for major depressive disorder globally without language restrictions using the search string (“depressive disorder”[Title/Abstract]) AND ((“service coverage”[Title/Abstract]) OR (“service gap”[Title/Abstract] OR “treatment coverage”[Title/Abstract] OR “treatment gap”[Title/Abstract])). This search sourced 44 publications, of which one study analysed the treatment coverage for major depressive disorder globally following a systematic review conducted in 2021. However, it did not specifically conduct a meta-regression analysis that estimated MAT for major depressive disorder by location, age, sex, and year. This was the most comprehensive review we came across on this topic.

**Added value of this study**

To our knowledge, this study is the first to quantify the extent to which children and adults with major depressive disorder accessed MAT globally as well as in 204 countries and territories, 21 regions, seven super-regions, by age and sex, between 2000 and 2021. From mental health surveys that assessed major depressive disorder within the general population as well as health services uptake by individuals with the disorder, we estimated MAT coverage among major depressive disorder cases by age, sex, location, and year. These estimates are designed to inform ongoing efforts to monitor response to the mental health needs of individuals with major depressive disorder globally. Work is already underway to incorporate our outputs within Global Burden of Disease analyses for major depressive disorder, the results framework of WHO's new general programme of work, and the Countdown for Global Mental Health 2030 initiative.

**Implications of all the available evidence**

The MAT rates estimated for major depressive disorder were very low in relation to the need or capacity to benefit from care, and the modest changes over time since 2000 fall short of a globally agreed target to increase service coverage for major depressive disorder by 50% by 2030. Urgent attention must be given to the scale-up of effective intervention strategies for major depressive disorder, especially in low-income and middle-income countries, as well as further research into better quality treatment options for this disorder.

Addressing the burden imposed by major depressive disorder requires a coordinated and multisectoral response to increase access to preventive and affordable treatment options, with established efficacy and for the necessary duration. With regard to treatment of major depressive disorder, clinical guidelines recommend a combination of pharmacotherapy (1 month of a medication, plus four visits to any type of medical doctor) or psychotherapy (eight visits with any professional) as the minimally adequate requirement for treatment.^5^, ^6^, ^7^, ^8^ Monitoring systems that track the extent to which mental health services in different countries adhere to these guidelines are limited by no or inconsistent reporting practices as well as an overall lack of data.^9^, ^10^

We planned to address this data shortage by developing a methodological framework that can maximise the use of available data on minimally adequate treatment (MAT) rates for major depressive disorder globally. To do this, we aimed to collate data from population mental health surveys on the services and treatment types accessed by those in the general population with major depressive disorder across the lifespan and to use established Bayesian meta-regression methodology developed as part of the GBD initiative to estimate rates of MAT by location, age, and sex from 2000 to 2021. These outputs could inform ongoing efforts to monitor response to the mental health needs of individuals at these locations. In particular, WHO's Comprehensive Mental Health Action Plan 2013–30 includes a specific target that “service coverage for mental health conditions will have increased at least by half, by 2030” and a related indicator, the “proportion of people with depression who are using services over the past 12 months (%)”.^11^ Furthermore, the Countdown for Global Mental Health 2030 initiative recommends and uses MAT rates for major depressive disorder (alongside other indicators) to monitor progress for mental health against Sustainable Development Goals (SDGs).^10^

## Methods

### Study design

For this study, data on major depressive disorder treatment coverage were sourced from systematic reviews, the GBD 2021 epidemiological dataset for major depressive disorder,^3^ and communication with experts. Following bias correction for known sources of measurement error between studies, these data informed a meta-regression model providing MAT coverage among people with major depressive disorder by age, sex, location, and year. Final estimates were standardised by age and sex against the GBD 2021^3^ age and sex distribution of people with major depressive disorder globally. We followed the GBD location hierarchy, which includes 204 countries and territories, 21 regions, and seven super-regions. A graphical overview of the analytical process is in the appendix (p 4). This study complies with GATHER (appendix pp 7–8). Ethics approval and participant consent were not sought. We used non-identifiable and pre-aggregated data from existing published or grey literature sources and no primary data collection was undertaken. People with lived experience were involved in the design, preparation, interpretation, and writing of this report.

### Case definition

We adhered to the definition of major depressive disorder proposed in the DSM-4-text revision, or the corresponding diagnosis of recurrent depression in the ICD-10 (diagnostic codes F32.0–9 and F33.0–9). Major depressive disorder is defined as an episodic mood disorder with at least one major depressive episode, characterised by depressed mood or loss of interest or pleasure for most of the day, every day, for 2 weeks.

Our definition of MAT was not preset and was guided by the available survey data. The majority of included studies adhered to the definition of MAT presented by Wang and colleagues^5^ derived from evidence-based guidelines^6^, ^7^, ^8^, ^12^ recommending either pharmacotherapy (1 month of a medication, plus four visits to any type of medical doctor) or psychotherapy (eight visits with any professional). The option to have four visits to a medical doctor for pharmacotherapy was derived from guidelines suggesting this is generally the amount required for medication assessment, initiation, and monitoring of treatment. The option for eight visits for psychotherapy was derived from literature indicating treatment efficacy with at least eight sessions. These definitions are discussed elsewhere.^5^, ^6^, ^7^, ^8^, ^12^

### Data sources

Treatment rates were sourced from existing systematic reviews and data sources provided by experts. Eligible studies reported treatment rates of either MAT, any mental health service coverage, or antidepressant use within a 12-month recall period or less among individuals with major depressive disorder sourced from a general population sample. We assumed treatment was sought at the point when participants were experiencing a major depressive episode within the recall period of the study. We re-extracted data from 18 studies meeting our inclusion criteria from a systematic review by Moitra and colleagues^12^ that searched PubMed and Embase for papers published between Jan 1, 2000, and Nov 26, 2021, reporting on the treatment coverage for people with major depressive disorder. Additionally, we reviewed surveys reporting the prevalence of major depressive disorder within existing GBD epidemiological datasets informed by systematic reviews covering between 1980 and 2022, enabling the inclusion of an additional six studies.^1^, ^3^, ^13^ Another eight studies were provided by experts. Information on location, age, sex, uncertainty, sample size, year, and case definition was extracted from eligible studies. We extracted the most detailed treatment rates reported by service type, age, and sex. Data by ethnicity were not available.

### Data preparation

We followed the GBD approach to addressing known sources of measurement error within epidemiological data,^3^ with two adjustments made to the data before analysis. First, to maximise data inclusion, estimates of antidepressant use and any mental health service use were adjusted towards the level of MAT estimates. This was achieved by matching estimates of MAT, antidepressant use, or any mental health service use on location, age, sex, and year. We identified 63 pairs of estimates. The difference between the pairs of logit-transformed treatment coverage estimates were inputs in a network meta-analysis conducted via meta-regression—Bayesian, regularised, and trimmed (MR-BRT).^3^, ^14^ MR-BRT estimates Gaussian mixed-effects models with random (Gaussian) effects by study, and allows for a trimmed maximum likelihood estimator to make measures of association more robust by excluding outliers based on their contributions to the likelihood function. Trimming was set to 5% of the data. Antidepressant use and any mental health service use were included as covariates and the Healthcare Access Quality Index (HAQI) was included as a modifier of the association with these covariates. The HAQI is informed by the mortality rate of 32 causes of death which should not, or rarely, occur in the presence of effective care, and has been found to be associated with MAT estimates of mental disorders.^15^ It spans between 0 (worst) and 100 (best), representing the first and 99th percentiles observed since 1990.^16^ The HAQI was centred at 100 for the bias correction analysis to quantify the bias in estimates of antidepressant use and use of any mental health services in the best-case scenario by setting an HAQI of 100 as the intercept. The modifiers of association allowed for HAQI-dependent corrections with decreasing health-care access quality. The final model for bias corrections was developed via backward elimination, in which the least impactful covariate was iteratively removed (unless its effect modifier remained statistically significant) until no improvement was seen in the Bayesian information criterion. Several scale transformations for the HAQI were tested, resulting in negligible improvements to model fit (appendix p 18). Transitivity was assessed via sensitivity analysis to inspect the robustness of the model coefficients with versus without indirect comparisons (appendix p 37). Pooled bias corrections by HAQI were extrapolated from this model and used to adjust estimates of antidepressant use and any mental health service use to reflect estimates of MAT coverage.

Second, estimates aggregated across sexes within the study sample were split into sex-specific estimates. For studies reporting granular data by age but only reporting sex-specific data across all ages, the age-specific data were split by the within-study sex ratio. Studies that did not report sex-specific data were split via a pooled adjustment. Bias-corrected MAT estimates informed a MR-BRT model with proportion of people with major depressive disorder who were female as the independent variable. Where studies did not report the proportion of people with major depressive disorder who were female, this information was taken from the estimated prevalence of major depressive disorder for the location-year of the estimate from GBD. Studies varied in the proportion of people with major depressive disorder (the denominator of the estimate) who were female. If MAT use varied by sex, then the required adjustment to these both-sex estimates would need to vary by the proportion of people with major depressive disorder who were female. We achieved this via a MR-BRT model with proportion of people with major depressive disorder who were female as the dependent variable and the intercept representing estimates where every person with major depressive disorder was male (ie, male-specific estimates). We then generated a prediction matrix to quantify how MAT use estimates vary depending on the proportion of people with major depressive disorder who were female for each estimate, and adjusted the both-sex data accordingly.

### Statistical analysis

Sex-specific estimates were used as inputs to estimate MAT coverage via the Bayesian meta-regression tool, DisMod-MR 2.1. This tool pools heterogeneous epidemiological data and generates modelled estimates for locations missing raw epidemiological data by drawing on estimates from surrounding locations and predictive covariates. This process is achieved by fitting estimates across the GBD location hierarchy: global, super-region, region, country, and subnational locations, with fits higher in geographical level acting as priors for lower geographical levels. DisMod-MR 2.1 allows for country-level covariates to inform estimates in locations without raw data and we included the HAQI as a country-level covariate. More detail on DisMod-MR 2.1 is described elsewhere.^17^ Age priors of 0% between the ages of 0 and 1 years, and a decreasing trend between ages 80 and 100 years were applied to guide DisMod-MR 2.1 following the age pattern in the data. A time window of 7 years was applied (meaning estimates would inform use 7 years before and after the estimate was collected) to remain consistent with the major depressive disorder DisMod-MR 2.1 model for GBD 2021. We projected MAT coverage estimates by age, sex, location, and year between 2000 and 2021. Out-of-sample validation was inspected via sensitivity analysis excluding data for 20% of countries with input data (appendix p 3).

Modelled MAT estimates were weighted by the estimated location, age, sex, and year-specific major depressive disorder prevalence from GBD 2021.^3^ The method used in GBD 2021 to estimate the prevalence of major depressive disorder is discussed elsewhere.^1^, ^3^, ^4^ In brief, first, the prevalence of major depressive disorder was estimated via a DisMod-MR 2.1 analysis of pre-2020 epidemiological data sourced from systematic reviews.^1^, ^3^, ^13^ Second, a MR-BRT model quantifying the association between the increase in prevalence of major depressive disorder and indicators of the impact of the COVID-19 pandemic (ie, human mobility and COVID-19 mortality) was used to extrapolate the increase in prevalence during the COVID-19 pandemic from the baseline prevalence estimated by DisMod-MR 2.1 for the years 2020 and 2021.^3^ In this study, we generated 500 samples of the estimated number of individuals with major depressive disorder by age and sex estimated by GBD 2021 using R (version 4.2.2).^18^ We then generated 500 samples of MAT treatment coverage estimated by DisMod-MR 2.1 by age, sex, location, and year and multiplied them by their respective estimates of the number of people with major depressive disorder and MAT. MAT treatment coverage by GBD region, super-region, and globally were calculated by aggregating across locations within each level of the location hierarchy. UIs represented the final 2·5th and 97·5th percentile values across the 500 samples and the point estimate represented the mean of the samples.

### Role of the funding source

The funders of the study had no role in study design, data collection, data analysis, data interpretation, or writing of the report.

## Results

**Figure 1:**
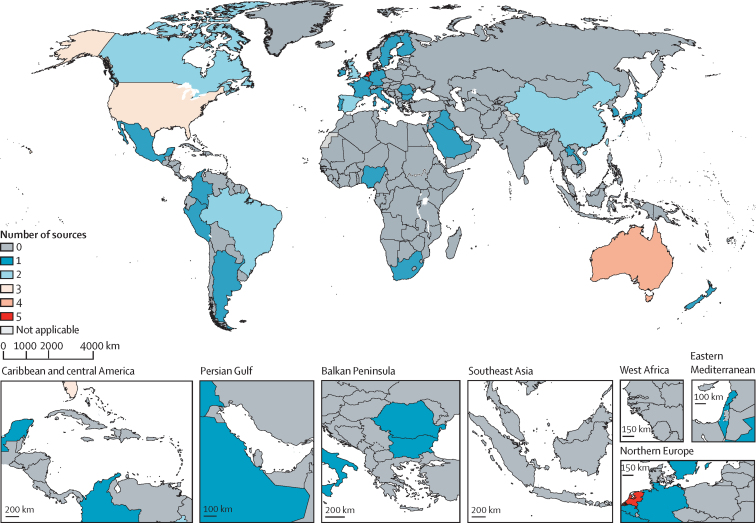
The number of sources per country used to inform the estimation of minimally adequate treatment The geographical delineations presented in this figure are in accordance with WHO standards and differ from the geographical hierarchy used in the statistical analyses. Dotted and dashed lines on maps represent approximate border lines for which there may not yet be full agreement.

The final dataset consisted of 145 estimates across 32 studies reporting on 45 population-representative surveys across 31 countries within 14 regions and six super-regions (figure 1). Six surveys reported sex-specific estimates of treatment coverage, 28 surveys reported estimates of MAT coverage, 30 reported coverage of antidepressant use, and 28 reported coverage of use of any mental health services (appendix pp 9–16).

The estimation of bias corrections suggested that estimates of the proportion of antidepressant use tended to over-estimate MAT treatment coverage (table 1). Estimates of any mental health service use did not differ considerably from estimates of MAT at an HAQI of 100 (where the HAQI was centred for analysis). However, the bias correction for estimates of any mental health service use among people with major depressive disorder varied by HAQI, with a greater gap between MAT coverage and any mental health service use for locations with a lower HAQI. Females with major depressive disorder were also more likely to receive MAT than males with major depressive disorder (table 1).

**Table 1:** Summary of bias corrections

	**Coefficient (95% UI)**	**Odds ratio (95% UI)**	**p value**
**Bias correction**			
Estimate represents any use of antidepressants	0·18 (0·07 to 0·29)	1·20 (1·07 to 1·33)	0·0010
Estimate represents any mental health service use	0·06 (−0·17 to 0·29)	1·06 (0·85 to 1·34)	0·59
Interaction between estimate represents any mental health service use and HAQI*	−0·012 (−0·024 to −0·001)	0·988 (0·977 to 0·999)	0·029
**Sex-specific analysis**			
Intercept	−1·74 (−1·95 to −1·52)	0·18 (0·14 to 0·22)	<0·0001
Proportion of people with major depressive disorder who are female, %	0·35 (0·17 to 0·53)	1·42 (1·18 to 1·70)	0·0001

HAQI=Healthcare access quality index. UI=uncertainty interval.

* HAQI mean centred at 100 and so primary association of mental health service use was not statistically significant at this high HAQI.

**Figure 2:**
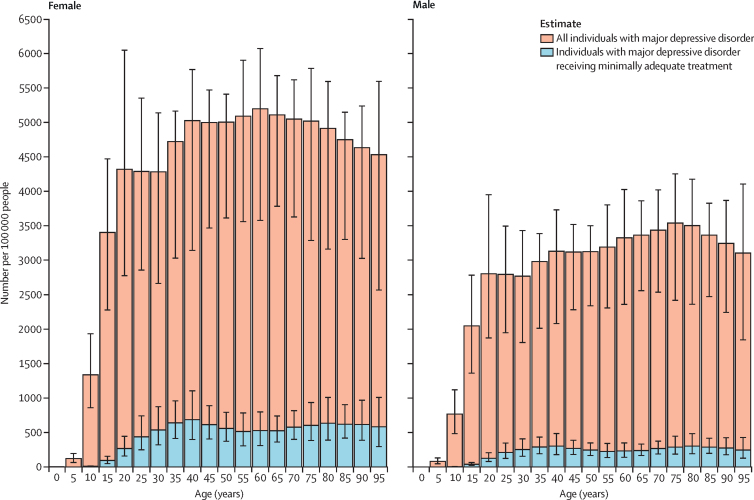
Global prevalence of major depressive disorder and rate of minimally adequate treatment coverage for major depressive disorder per 100 000 people by sex, 2021 Whiskers show 95% uncertainty intervals.

The estimated global coverage of MAT among people with major depressive disorder in 2021 was 9·1% (95% UI 7·2–11·6). Globally, a larger proportion of females with major depressive disorder received MAT (10·2% [8·1–13·1]) than males with major depressive disorder (7·2% [5·7–9·3]). MAT coverage for people with major depressive disorder increased steadily from age 15 years up to age 40 years and then remained stable across the remaining lifespan (figure 2).

**Figure 3:**
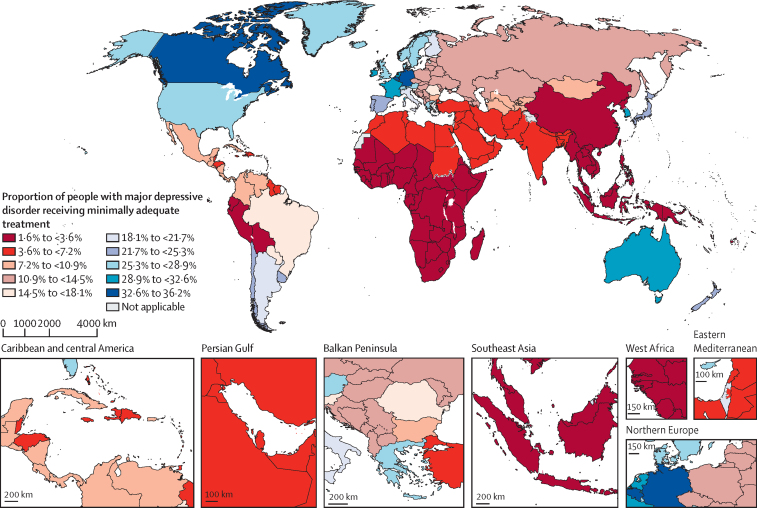
The proportion of people with major depressive disorder receiving minimally adequate treatment by country, 2021 The geographical delineations presented in this figure are in accordance with WHO standards and differ from the geographical hierarchy used in the statistical analyses. Dotted and dashed lines on maps represent approximate border lines for which there may not yet be full agreement.

We estimated the distribution of MAT coverage by country, region, super-region, and sex for 2021 (figure 3 and table 2) and further summarised these estimates by location (country, region, and subregion) and sex for 2000 and 2021 (appendix pp 19–27). The super-region with the largest MAT coverage was high-income at 27·0% (95% UI 21·7–34·4). Within this super-region, the region with the highest MAT coverage was Australasia at 29·2% (21·4–40·8), and the country with the highest MAT coverage was Canada at 36·2% (26·1–50·0; figure 3). The super-region with the lowest MAT coverage was sub-Saharan Africa at 2·0% (1·5–2·6), and the region with the lowest MAT coverage was western sub-Saharan Africa at 1·8% (1·4–2·5), with minimal variation in estimates across countries within this region.

**Table 2:** Proportion of people with major depressive disorder receiving minimally adequate treatment by sex, super-region, and region, 2021

		**Total (95% UI)**	**Females (95% UI)**	**Males (95% UI)**
Global		9·1% (7·2–11·6)	10·2% (8·2–13·1)	7·2% (5·7–9·3)
Central Europe, eastern Europe, and central Asia		12·6% (10·1–16·1)	13·9% (11·1–17·8)	10·3% (8·2–13·2)
	Central Asia	10·8% (7·8–15·2)	12·0% (8·8–16·6)	8·7% (6·1–12·6)
	Central Europe	13·6% (10·4–18·2)	14·7% (11·1–19·7)	10·9% (8·4–14·4)
	Eastern Europe	12·8% (10·6–15·8)	14·2% (11·8–17·5)	10·5% (8·7–13·0)
High-income		27·0% (21·7–34·4)	29·0% (23·4–36·8)	23·4% (18·7–30·3)
	Australasia	29·2% (21·4–40·8)	31·9% (23·2–44·6)	25·2% (18·2–35·8)
	High-income Asia–Pacific	26·4% (21·9–32·4)	29·1% (24·3–35·5)	22·0% (18·2–27·4)
	High-income North America	27·8% (22·8–34·3)	29·6% (24·2–36·6)	24·3% (20·1–29·9)
	Southern Latin America	22·4% (16·0–31·6)	24·1% (17·4–33·9)	19·1% (13·5–26·9)
	Western Europe	26·9% (20·9–35·7)	28·9% (22·4–38·1)	23·2% (18·0–31·5)
Latin America and Caribbean		10·8% (8·8–13·6)	11·9% (9·6–15·0)	8·4% (6·8–10·6)
	Andean Latin America	2·0% (1·4–2·9)	2·2% (1·5–3·2)	1·7% (1·2–2·5)
	Caribbean	6·6% (4·8–9·4)	7·2% (5·2–10·3)	5·6% (4·0–7·9)
	Central Latin America	7·7% (6·1–9·8)	8·4% (6·6–10·8)	6·2% (4·9–8·1)
	Tropical Latin America	16·0% (13·1–19·9)	17·3% (14·1–21·6)	12·7% (10·5–15·8)
North Africa and Middle East		5·2% (3·6–7·3)	5·7% (4·1–8·1)	4·3% (3·0–6·1)
South Asia		5·7% (4·6–7·2)	6·4% (5·2–8·0)	4·8% (3·9–6·0)
Southeast Asia, east Asia, and Oceania		2·3% (1·9–2·9)	2·6% (2·1–3·2)	1·9% (1·5–2·4)
	East Asia	2·5% (2·0–3·0)	2·7% (2·2–3·4)	2·0% (1·6–2·4)
	Oceania	1·7% (1·1–2·6)	2·0% (1·3–2·9)	1·5% (0·9–2·3)
	Southeast Asia	2·0% (1·6–2·7)	2·2% (1·7–3·0)	1·7% (1·3–2·3)
Sub-Saharan Africa		2·0% (1·5–2·6)	2·2% (1·6–2·9)	1·7% (1·2–2·2)
	Central sub-Saharan Africa	1·9% (1·2–2·9)	2·2% (1·4–3·3)	1·6% (1·0–2·4)
	Eastern sub-Saharan Africa	2·0% (1·5–2·6)	2·2% (1·7–3·0)	1·6% (1·2–2·1)
	Southern sub-Saharan Africa	2·6% (2·1–3·3)	3·3% (2·7–4·3)	1·4% (1·1–1·8)
	Western sub-Saharan Africa	1·8% (1·4–2·5)	1·8% (1·3–2·4)	1·9% (1·5–2·6)

UI=uncertainty interval.

Only seven countries, all from high-income settings (Australia, Belgium, Canada, Germany, South Korea, Sweden, and the Netherlands), had MAT rates that exceeded 30% of individuals with major depressive disorder in the population. By comparison, 90 countries from the rest of the world had a MAT rate of less than 5% of individuals with major depressive disorder in the population. 47 countries across all income groups had MAT rates between 5% and 9% of individuals with major depressive disorder in the population (appendix pp 19–27).

Globally, MAT coverage did not change between 2000 and 2021 (table 3). However, when estimates were disaggregated by GBD super-region, MAT coverage in all GBD regions increased in an example of Simpson's paradox,^19^ whereby aggregating the data across GBD regions and super-regions to calculate the global coverage hides the improvements within each region due to differences in the number of people with major depressive disorder by geography and time (table 3). The GBD super-region with the largest increase in MAT use was north Africa and the Middle East; the two GBD super-regions with the smallest increases in MAT use were high-income and Latin America and the Caribbean (table 3). Inspection of UIs suggests the increase in estimated MAT use in north Africa and the Middle East was larger than in central Europe, eastern Europe, and central Asia, high-income, Latin American and the Caribbean, and sub-Saharan Africa (table 3). Changes in MAT coverage by country, region, and super-region between 2000 and 2021 are shown in the appendix (pp 28–36).

**Table 3:** Counts and proportions of people with major depressive disorder receiving minimally adequate treatment in 2000 and 2021 by super-region

	**2000**			**2021**			**Change in MAT use (95% UI), %**
	People with major depressive disorder (95% UI), millions	Number receiving MAT (95% UI), millions	Proportion receiving MAT (95% UI), %	People with major depressive disorder (95% UI), millions	Number receiving MAT (95% UI), millions	Proportion receiving MAT (95% UI), %	
Global	149·2 (133·1–172·8)	13·7 (10·7–17·6)	9·2% (7·4–11·7)	234·4 (204·9–275·7)	21·3 (16·3–27·8)	9·1% (7·2–11·6)	−1·2% (−4·6 to 2·0)
Central Europe, eastern Europe, and central Asia	11·0 (9·8–12·6)	1·2 (1·0–1·6)	11·3% (9·1–14·3)	13·1 (11·3–15·2)	1·7 (1·3–2·2)	12·6% (10·1–16·1)	11·7% (9·8 to 13·5)
High-income	30·8 (28·1–34·5)	7·9 (6·3–10·1)	25·7% (20·7–32·7)	41·2 (36·8–47·9)	11·1 (8·6–14·6)	27·0% (21·7–34·4)	5·3% (1·7 to 9·2)
Latin America and the Caribbean	12·0 (10·6–13·9)	1·2 (1·0–1·6)	10·1% (8·2–12·8)	19·8 (17·2–23·3)	2·1 (1·6–2·8)	10·8% (8·8–13·6)	6·7% (2·3 to 11·8)
North Africa and Middle East	12·3 (10·8–14·6)	0·5 (0·4–0·8)	4·3% (3·0–6·1)	24·4 (20·0–29·6)	1·3 (0·8–1·9)	5·2% (3·6–7·3)	19·8% (14·1 to 25·0)
South Asia	36·9 (32·1–43·4)	1·9 (1·4–2·5)	5·1% (4·1–6·4)	60·2 (51·8–71·1)	3·4 (2·7–4·5)	5·7% (4·6–7·2)	12·0% (9·3 to 15·2)
Southeast Asia, east Asia, and Oceania	27·4 (24·5–31·6)	0·6 (0·4–0·7)	2·1% (1·7–2·6)	38·9 (33·8–45·1)	0·9 (0·7–1·2)	2·3% (1·9–2·9)	12·8% (4·3 to 21·4)
Sub-Saharan Africa	18·8 (16·2–22·2)	0·3 (0·3–0·5)	1·8% (1·4–2·4)	36·7 (31·0–44·0)	0·7 (0·5–1·0)	2·0% (1·5–2·6)	7·1% (5·1 to 9·4)

MAT=minimally adequate treatment. UI=uncertainty interval.

## Discussion

In 2021, after more than 30 years of advocacy and a decade of high-level political commitment for urgent attention to be given to the burden imposed by major depressive disorder,^11^ our results suggest that the global MAT gap for major depressive disorder was 90·9%. Here we present a means by which the MAT gap for major depressive disorder can be quantified and monitored over time. We hope this might generate more precise action from the global health community and other mental health stakeholders as they respond to the major depressive disorder burden.

Our MAT estimates provide a baseline from which progress made by countries to improve services for people with major depressive disorder can be measured. Globally, females were more likely to receive MAT than males. Those aged 40 years and older were also more likely to receive services, leaving larger treatment gaps for younger, more vulnerable populations. We observed considerable disparity in MAT access globally, with access ranging between 1% and 3% in many low-income and middle-income countries situated in sub-Saharan Africa and Asia in 2021. Even in the best of cases, the treatment gap was concerning, with MAT coverage ranging between 19·5% (95% UI 16·6–23·4) and 36·2% (26·1–50·0) in countries in the high-income region in 2021. Although modest relative increases in MAT since 2000 for each region were observed, countries across all income groups have been unable to substantially close the major depressive disorder treatment gap.^2^, ^10^

WHO's Comprehensive Mental Health Action Plan 2013–30 includes a target that service coverage for depression be increased by at least 50% by 2030*.*^10^ This requires substantial investment in scaling up of effective intervention strategies for major depressive disorder. There is increasing evidence attesting to the cost-effectiveness and returns on investment of depression treatment.^20^, ^21^, ^22^ The expected returns on this investment from improved health and labour force participation resulting from scaled-up depression treatment outweighed costs by 2·3–3·0 to 1 for economic benefits and 3·3–5·7 to 1 when the value of health returns was also considered.^21^ Similar analyses investigating lifelong impacts of addressing anxiety, depression, bipolar disorder, and suicide in young people aged 10–19 years across 36 countries estimated a return of US$24 in health and economic benefits to the economy over 80 years, for each $1 spent on the required interventions.^22^, ^23^

One reason for low major depressive disorder service coverage is the scarcity of mental health workforce, including specialists, especially in low-income and middle-income countries.^9^, ^24^ Guidelines for the effective treatment of depression in non-specialised settings are available and have been adopted by many low-income and middle-income countries, for example via training in WHO's Mental Health Gap Action Programme intervention guide.^11^, ^20^ Other reasons for stagnation include the influence of stigma and discrimination on help-seeking behaviours,^25^ challenges health-care providers face in detecting depression, and low availability or under-supply of psychological therapies and medication.^9^ Studies have shown how such systemic challenges or service barriers can be overcome by integrating depression treatment into national health plans, defined benefit packages, and financial protection schemes, as well as primary health-care delivery.^24^

We estimated MAT rates in 2021, when the impact of the COVID-19 pandemic should not be overlooked. We identified only two studies conducted during 2020–21, which suggested that although major depressive disorder prevalence might have increased during this time,^4^ MAT rates remained unchanged.^26^, ^27^ Our findings suggest that against a general increase in MAT coverage since 2000, the pandemic period of 2020–21 exerted little separable effect on MAT coverage rates for major depressive disorder. However, if the pandemic caused a global change in MAT rates not captured here, based on the available literature of how mental health services were impacted during the worst phases of the pandemic, such a change is likely to have been a decrease in MAT rates. Alternative data sources have indicated that during the worst phases of the pandemic resources needed to be redirected to an emergency COVID-19 response, mental health service uptake decreased even when the incidence of major depressive disorder increased, and physical distancing or restricted travel mandates made it challenging to access treatment or medication.^4^, ^28^

There are several limitations and caveats to our estimates that need to be considered. First, estimates of MAT from low-income and middle-income locations, stratified by year, sex, and age, were limited by the availability of population representative mental health surveys and also by considerable heterogeneity across studies in how treatment access and MAT were operationalised. It is important to consider this limitation when interpreting the effect sizes and trends within the estimated rates of MAT. We adopted the broader approach used in GBD analyses, in which producing an uncertain estimate is preferable to the alternative of having no estimate at all.^1^, ^3^ For comparison, the size and coverage of the dataset informing our MAT estimates are similar to those of the datasets informing the prevalence estimation of several mental disorders in GBD 2021. Making MAT data available by location, age, sex, and year (albeit within large bounds of uncertainty) enables treatment rates of major depressive disorder to be considered alongside other indicators within international monitoring efforts to improve government response to the health needs of their populations.

Second, the definition of MAT used should be considered in future data collection efforts. Our analysis relied on the definition of MAT used within pre-aggregated data from our literature review. Most reported estimates of MAT (24 of 27 datapoints) came from surveys defining MAT as either pharmacotherapy (1 month of a medication, plus four visits to any type of medical doctor) or psychotherapy (eight visits with any professional). However, there is a debate in the broader literature regarding best practice for treating major depressive disorder, such as what should be considered as minimally adequate pharmacotherapy or whether non-pharmacological physical treatment options such as light therapy or neurostimulation should be incorporated.^29^

Third, many of the studies informing our estimates of MAT represented estimates of antidepressant use or any mental health service use because these two forms of estimates were considered close proxies to MAT compared with other service use estimates that might be available (eg, any service use). We adjusted these estimates to reflect likely MAT before modelling; however, owing to scarce data, our correction could not be sex-specific. Fourth, we used the HAQI to adjust estimates of antidepressant use and any mental health service use, and more broadly as a location-level covariate in our DisMod-MR 2.1 model. The HAQI is estimated using mortality from preventable causes.^16^ This reliance on the HAQI probably masked some of the true variation in MAT across locations because there would still be variation in mental health service availability across countries with similar HAQI scores depending on the country-specific policy and funding priorities. Fifth, the UIs reported do not consider biases that could not be quantified, such as selection bias, model misspecification bias, and uncaptured measurement bias. Sixth, our estimates of MAT coverage used modelled prevalence estimates for major depressive disorder for GBD 2021, which are affected by similar limitations and have been discussed in detail elsewhere.^1^, ^3^

Seventh, studies included in this analysis measured service use within prevalent cases of major depressive disorder over a 12-month period. It was our assumption that treatment was sought at the point when participants were experiencing a major depressive episode within that recall period; however, it was not possible to verify this with the available data. Last, estimates of MAT could not distinguish between location, age, and sex-specific trends in service access versus service completion, nor could they distinguish between individuals opting not to engage with services (despite services being available) and those who could not engage due to lack of available services. A separate analysis of barriers to accessing mental health care among individuals with common mental disorders identified low perceived need, attitudinal barriers (for those experiencing symptoms of low to moderate severity), and broader systemic barriers (for those experiencing severe symptoms) as key challenges to seeking and remaining in treatment globally.^30^

Our modelled estimates of MAT for major depressive disorder have several applications. They have been incorporated within the Countdown for Global Mental Health 2030 initiative (alongside other indicators) to monitor progress for mental health against SDGs.^31^ Additionally, our findings could inform a newly agreed outcome in WHO's 14th Global Programme of Work and Results Framework to “increase service coverage for mental health and neurological conditions”.^32^ Our estimates of MAT are also included in upcoming GBD analyses of major depressive disorder burden to better quantify variation in the severity of major depressive disorder by health-care access.^1^, ^3^

There are several opportunities for future research to improve on our analyses. Upcoming mental health surveys should include more detailed estimates of MAT within their scope so that the global data coverage might be improved. New data collection efforts should take into consideration updates made to clinical guidelines and best practices for treating major depressive disorder and how these change across the lifespan. We hope that case definitions and inclusion criteria adhered to in this study can guide future data collection efforts. With additional data across place and time, our estimates of MAT can become more precise and globally representative, aiding systemicatic monitoring of the scale-up of services.

More generally, research into efficacious treatment options for major depressive disorder remains of the utmost priority. Although the type and availability of services have improved over the past four decades, there has been no detectable decline in major depressive disorder prevalence at the population level.^33^, ^34^ Proposed explanations have included the possibility that a decrease in prevalence is offset by an increase in incidence (either as a true increase of new cases or increases due to misdiagnosis). Other explanations relate to lower quality of treatment for major depressive disorder offsetting a decline in prevalence. Treatment options might be less efficacious than indicated, effect sizes achieved within clinical trials cannot always be generalised to real-world applications, treatment might have greater impact for individuals with chronic and recurrent presentations of major depressive disorder compared with non-recurrent presentations, and treatment might cause side-effects leading to iatrogenic consequences.

In 2021, the MAT rates for major depressive disorder were far from commensurate to the mental health need globally. Urgent attention should be given to the scale-up of effective intervention strategies, especially in low-income and middle-income countries. Guidelines for how this can be achieved have been well articulated.^11^, ^20^ Further research should prioritise better quality treatment options for major depressive disorder. We hope that our findings can generate more precise action and accountability from the global health community, governments, and other mental health stakeholders as we uphold the right to mental health for all.

### Contributors

### Data sharing

To download code and data used in these analyses, please visit the GitHub page http://github.com/ihmeuw/mental_disorders/tree/mdd_mat.

**This online publication has been corrected. The corrected version first appeared at thelancet.com/psychiatry on November 19, 2024**

## Declaration of interests

SS is a senior advisor to McKinsey Health Institute. All other authors declare no competing interests.
