# Correction to *Lancet Psychiatry* 2024; 11: 1012–21

**DOI:** 10.1016/S2215-0366(24)00398-5

**Published:** 2025-01

*Santomauro DF, Vos T, Whiteford HA, Chisholm D, Saxena S, Ferrari AJ. Service coverage for major depressive disorder: estimated rates of minimally adequate treatment for 204 countries and territories in 2021.* Lancet Psychiatry *2024;* 11: *1012–21*—In figures 1 and 3 of this Article, the colours for some locations have been updated and statements added to the legends regarding WHO standards for geographical delineations. In the appendix, the names of some locations have been updated. These corrections have been made to the online version as of Nov 19, 2024.

