# Supplementary material for: Service coverage for major depressive disorder: estimated rates of minimally adequate treatment for 204 countries and territories in 2021
Source: Lancet Psychiatry. 2024 Dec;11(12):1012–21. doi: 10.1016/S2215-0366(24)00317-1 (PMC11579305; doi:10.1016/S2215-0366(24)00317-1)
Supplement: Supplementary appendix [file mmc1.pdf]

THE LANCET
Psychiatry

Supplementary appendix

This appendix formed part of the original submission and has been peer reviewed. We post it as supplied by the authors.

Supplement to: Santomauro DF, Vos T, Whiteford HA, Chisholm D, Saxena S, Ferrari AJ. Service coverage for major depressive disorder: estimated rates of minimally adequate treatment for 204 countries and territories in 2021. *Lancet Psychiatry* 2024; **11:** 1012–21.

Contents

List of figures and tables 2
Figures 2
Tables 2
Section 1. Supplementary methods 3
Section 1.1 Assessment of transitivity of the bias correction model 3
Section 1.2 Out-of-sample validation 3
Section 2. Figures and tables 4
Figure S1: Overview of the estimation of minimally adequate treatment utilisation among persons with major depressive disorder 4
Figure S2: Out-of-sample validation of random 20% of countries with data excluded, females 5
Figure S3: Out-of-sample validation of random 20% of countries with data excluded, males 6
Table S1: Guidelines for Accurate and Transparent Health Estimates Reporting (GATHER) checklist 7
Table S2: Characteristics of included studies 9
Table S3: Candidate models for the estimation of bias corrections for non-reference data 17
Table S4: Candidate scale transformations for the HAQI in the estimation of bias corrections for non-reference data 18
Table S5: Proportion of persons with major depressive disorder receiving minimally adequate treatment by sex and location for 2000 and 2021 (95% UIs). 19
Table S6: Counts and proportions of major depressive disorder cases receiving minimally adequate treatment in 2000 and 2021 by location (95% UIs) 28
Table S7: Sensitivity analysis to assess the impact of indirect effects on the bias correction model 37

List of figures and tables

Figures

Figure S1: Overview of the estimation of minimally adequate treatment utilisation among persons with major depressive disorder

Figure S2: Out-of-sample validation of random 20% of countries with data excluded, females

Figure S3: Out-of-sample validation of random 20% of countries with data excluded, males

Tables

Table S1: Guidelines for Accurate and Transparent Health Estimates Reporting (GATHER) checklist

Table S2: Characteristics of included studies

Table S3: Candidate models for the estimation of bias corrections for non-reference data

Table S4: Candidate scale transformations for the HAQI in the estimation of bias corrections for non-reference data

Table S5: Proportion of persons with major depressive disorder receiving minimally adequate treatment by sex and location for 2000 and 2021 (95% UIs).

Table S6: Counts and proportions of major depressive disorder cases receiving minimally adequate treatment in 2000 and 2021 by location (95% UIs)

Section 1. Supplementary methods

Section 1.1 Assessment of transitivity of the bias correction model

To assess assumptions of transitivity for the network meta-regression in the bias correction model, we conducted a sensitivity analysis by re-conducting this analysis after removing the 19% of input data that were indirect comparisons and checking the robustness of the coefficients. Final betas of this analysis were very similar after excluding indirect comparisons, however with wider uncertainty due to the reduced dataset (Table S7).

Section 1.2 Out-of-sample validation

To inspect how well this model estimates data for locations with missing data, we conducted a model excluding all data for 20% of randomly selected countries covered by the dataset. Random selection was conducted via the *sample* function within R without replacement. Countries selected at random were Belgium, Ireland, Lebanon, New Zealand, Portugal, Romania, and South Africa. Inspection of age-sex-year specific estimates for these countries illustrated little variation in modelled estimates with and without this data removed (Figure S2 and S3). Where there was variation, (e.g., Portugal and Romania), final estimates where well within the uncertainty intervals of the input data for those countries.

Section 2. Figures and tables

Figure S1: Overview of the estimation of minimally adequate treatment utilisation among persons with major depressive disorder

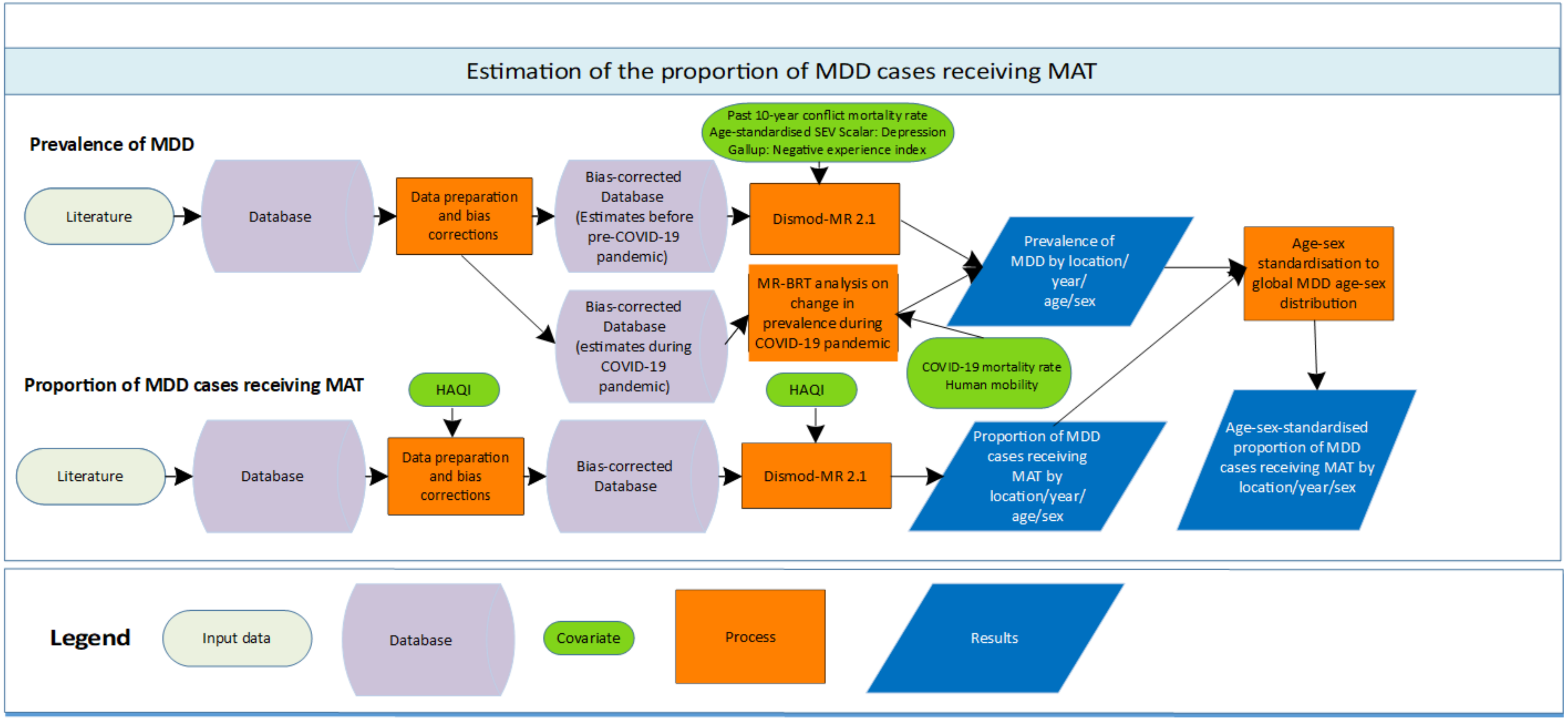

Note: Bias corrections applied to MDD prevalence data included adjustments to past-year prevalence estimates (to reflect point prevalence), estimates relying on lay interviewers (vs clinicians), estimates using symptom scales (vs diagnostic interviews), and to World Health Survey data (vs DSM-IV diagnostic criteria). Bias corrections applied to estimates of MAT included adjustments to any mental health service use (vs MAT) and antidepressant use (vs MAT). HAQI = Healthcare access quality index. MDD = Major depressive disorder. MAT = Minimally adequate treatment. SEV = Summary exposure value.

Figure S2: Out-of-sample validation of random 20% of countries with data excluded, females

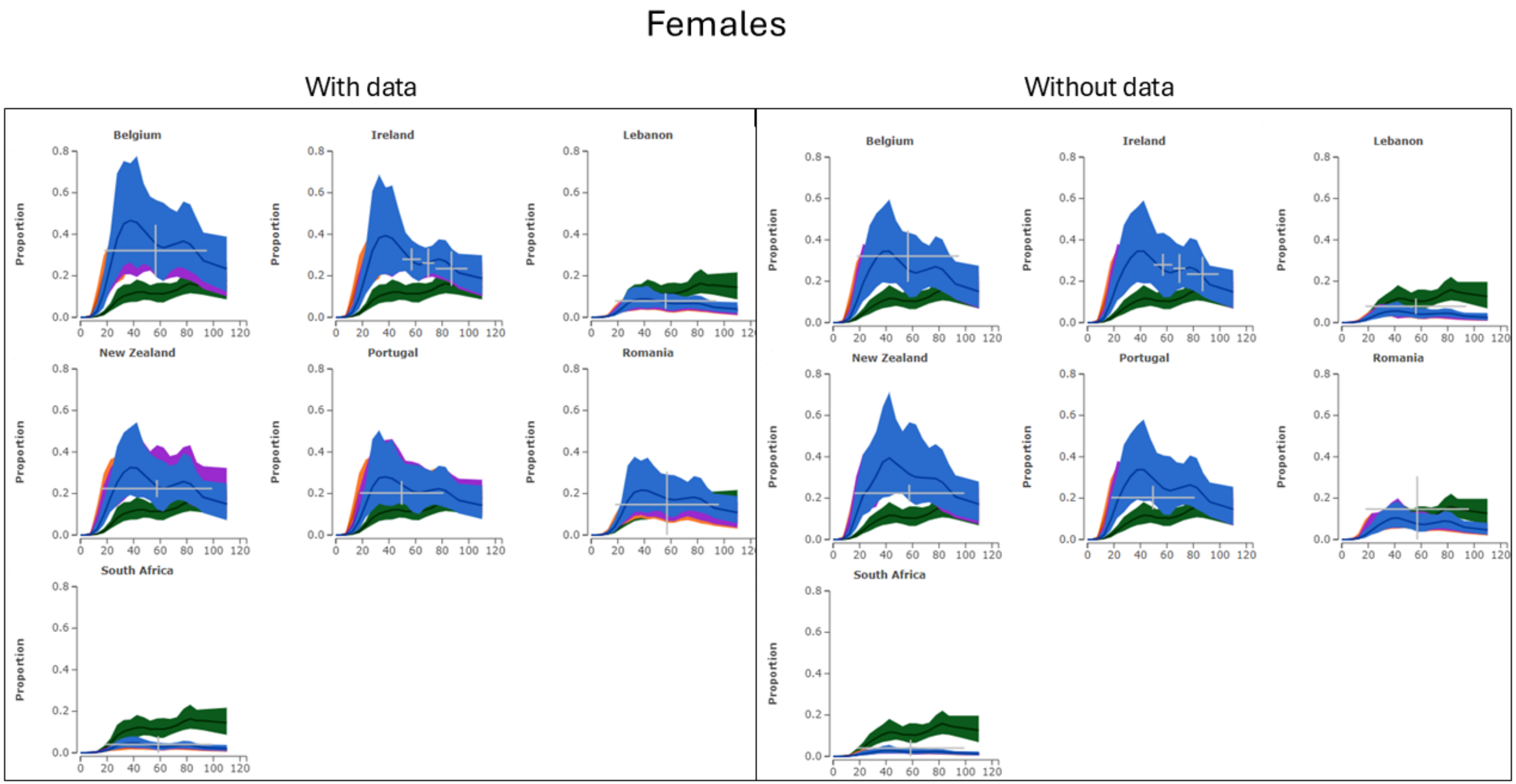

Plots in EpiViz for the year 2005 which captured all input data within the DisMod-MR 2.1 time window. Grey crosses represent input data, with the horizontal line representing the age range of the estimate and the vertical line representing the 95% uncertainty interval (UI) around the estimate. Input data shown in both plots but only used in plot labelled “With data”. The blue line represents the modelled estimated minimally adequate treatment utilisation among major depressive disorder (MDD) cases by country, with the blue area representing the 95% UI around the estimate. The purple area represents the 95% UI around the GBD region estimate. The orange area represents the 95% UI around the GBD super region estimate. The green line represents the global estimate and the green area represents the 95% uncertainty interval around the global estimate. Estimates not yet standardised by global MDD age-sex distribution.

Figure S3: Out-of-sample validation of random 20% of countries with data excluded, males

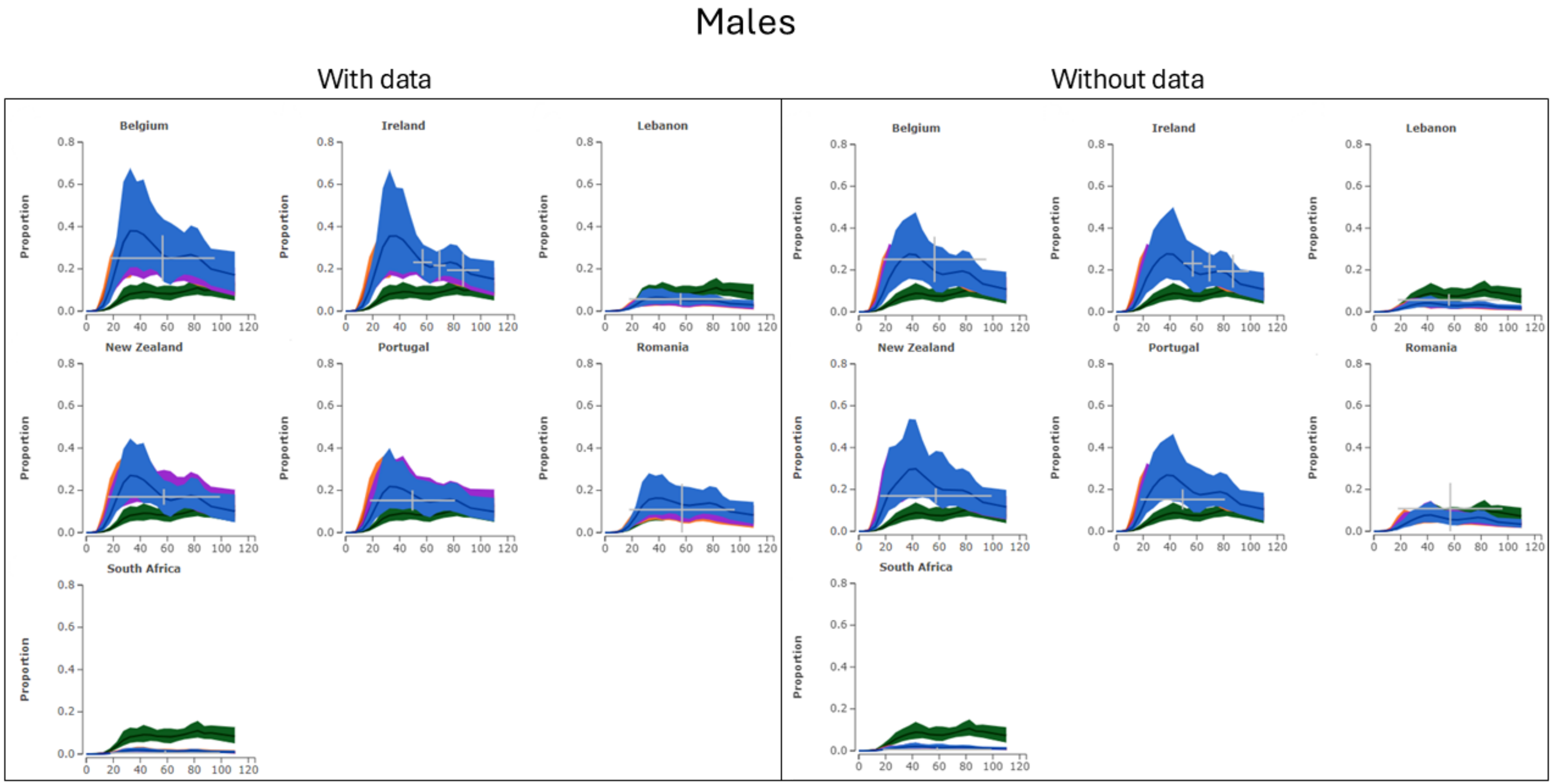

Plots in EpiViz for the year 2005 which captured all input data within the DisMod-MR 2.1 time window. Grey crosses represent input data, with the horizontal line representing the age range of the estimate and the vertical line representing the 95% uncertainty interval (UI) around the estimate. Input data shown in both plots but only used in plot labelled “With data”. The blue line represents the modelled estimated minimally adequate treatment utilisation among major depressive disorder (MDD) cases by country, with the blue area representing the 95% UI around the estimate. The purple area represents the 95% UI around the GBD region estimate. The orange area represents the 95% UI around the GBD super region estimate. The green line represents the global estimate and the green area represents the 95% uncertainty interval around the global estimate. Estimates not yet standardised by global MDD age-sex distribution.

Table S1: Guidelines for Accurate and Transparent Health Estimates Reporting (GATHER) checklist

Item #	Checklist item	Location
**Objectives and funding**		
1	Define the indicator(s), populations (including age, sex, and geographic entities), and time period(s) for which estimates were made.	Method: Case definition
2	List the funding sources for the work.	Acknowledgements
**Data Inputs**		
For all data inputs from multiple sources that are synthesized as part of the study:		
3	Describe how the data were identified and how the data were accessed.	Method: Data sources
4	Specify the inclusion and exclusion criteria. Identify all ad-hoc exclusions.	Method: Data sources
5	Provide information on all included data sources and their main characteristics. For each data source used, report reference information or contact name/institution, population represented, data collection method, year(s) of data collection, sex and age range, diagnostic criteria or measurement method, and sample size, as relevant.	Table S2
6	Identify and describe any categories of input data that have potentially important biases (e.g., based on characteristics listed in item 5).	Method: Data preparation
For data inputs that contribute to the analysis but were not synthesized as part of the study:		
7	Describe and give sources for any other data inputs.	Method: Data sources
For all data inputs:		
8	Provide all data inputs in a file format from which data can be efficiently extracted (e.g., a spreadsheet rather than a PDF), including all relevant meta-data listed in item 5. For any data inputs that cannot be shared because of ethical or legal reasons, such as third-party ownership, provide a contact name or the name of the institution that retains the right to the data.	Table S5<br>Table S6
**Data analysis**		
9	Provide a conceptual overview of the data analysis method. A diagram may be helpful.	Method: Overview<br>Figure S1
10	Provide a detailed description of all steps of the analysis, including mathematical formulae. This description should cover, as relevant, data cleaning, data pre-processing, data adjustments and weighting of data sources, and mathematical or statistical model(s).	Method: Data preparation<br>Method: Statistical analysis

11	Describe how candidate models were evaluated and how the final model(s) were selected.	Method: Statistical analysis
12	Provide the results of an evaluation of model performance, if done, as well as the results of any relevant sensitivity analysis.	Method: Statistical analysis<br>Appendix Section 2<br>Figure S2<br>Figure S3<br>Table S3<br>Table S7
13	Describe methods for calculating uncertainty of the estimates. State which sources of uncertainty were, and were not, accounted for in the uncertainty analysis.	Method: Statistical analysis
14	State how analytic or statistical source code used to generate estimates can be accessed.	*https://github.com/ihmeuw/mental_disorders/tree/mdd_mat*
**Results and Discussion**		
15	Provide published estimates in a file format from which data can be efficiently extracted.	*Table S5*<br>*Table S6*
16	Report a quantitative measure of the uncertainty of the estimates (e.g. uncertainty intervals).	All results provided with 95% uncertainty intervals
17	Interpret results in light of existing evidence. If updating a previous set of estimates, describe the reasons for changes in estimates.	Discussion: Significance of this work
18	Discuss limitations of the estimates. Include a discussion of any modelling assumptions or data limitations that affect interpretation of the estimates.	Discussion: Limitations

Table S2: Characteristics of included studies

Citation	Recall	Service type	Location	Years	Sex	Female cases (%)	Ages (years)	Treatment utilisation (%)	Lower CI (%)	Upper CI (%)	Total cases	Treated cases
Charlson F, Choulamany C, Diminic S, Santomauro D, Raja S, Whiteford H. The prevalence of mental and substance use disorders in Lao PDR: findings from a cross-sectional survey. [Forthcoming]	12 months	Any mental health service	Lao People's Democratic Republic	2015 to 2015	Both	56.2	18 to 99	0	0	14.8	13	0
Al-Habeeb A, Altwaijri YA, Al-Subaie AS, Bilal L, Almeharish A, Sampson NA, Liu H, Kessler RC. Twelve-month treatment of mental disorders in the Saudi National Mental Health Survey. Int J Methods Psychiatr Res. 2020; 29(3): e1832.	12 months	Any mental health service	Saudi Arabia	2011 to 2016	Both	Not reported	15 to 65	6.1	0.6	11.6	251	15
Australian Bureau of Statistics, Department of Health and Family Services (Australia). Australia National Survey of Mental Health and Wellbeing 1997.	12 months	Any mental health service	Australia	1997 to 1997	Both	66.3	16 to 99	32.3	27.9	37	762	271
					Female	100	16 to 34	37.5	28.1	47.9	204	82
					Female	100	16 to 99	33.2	27.6	39.2	528	187
					Female	100	35 to 44	36.8	27.5	47.1	136	54
					Female	100	45 to 54	35.2	24	48.3	103	36
					Female	100	55 to 99	14.7	8.1	25.3	85	15
					Male	0	16 to 34	29.1	17.3	44.6	80	30
					Male	0	16 to 99	30.5	23.4	38.7	234	84
					Male	0	35 to 44	31.8	20.6	45.6	65	24
					Male	0	45 to 54	35.4	19.1	56	53	20
					Male	0	55 to 99	24.6	8.8	52.3	36	10
		Minimally adequate treatment			Both	66.3	16 to 99	28.9	24.8	33	762	243
					Female	100	16 to 34	24.8	19.2	31.4	204	56
					Female	100	16 to 99	30.1	25.3	35.5	528	174
					Female	100	35 to 44	37.4	28	47.8	136	53
					Female	100	45 to 54	35.1	26.5	44.8	103	39
					Female	100	55 to 99	26.7	17.1	39.2	85	26
					Male	0	16 to 34	24.5	14	39.4	80	23
					Male	0	16 to 99	26.6	19.7	34.8	234	69

					Male	0	35 to 44	22.8	12.4	38.1	65	15
					Male	0	45 to 54	39.2	26.9	52.9	53	21
	12 months	Minimally adequate treatment	Australia	1997 to 1997	Male	0	55 to 99	19.6	8.5	38.8	36	10
Australian Bureau of Statistics. Australia National Survey of Mental Health and Wellbeing 2007. Canberra, Australia: Australian Bureau of Statistics, 2007.	Point	Antidepressants	Australia	2007 to 2007	Both	62.2	16 to 85	28.6	20.3	39	178	56
					Female	100	16 to 85	33.5	22.2	47	113	43
					Male	0	16 to 85	22.3	9.1	45.2	65	13
	12 months	Any mental health service			Both	62.2	16 to 85	37.4	31.1	44	442	160
					Female	100	16 to 34	37.3	27.7	48	106	37
					Female	100	16 to 85	38.4	31.2	46.2	293	113
					Female	100	35 to 44	40	23.8	58.7	62	30
					Female	100	45 to 54	42.9	21.3	67.7	49	22
					Female	100	55 to 85	33.6	20.9	49.2	76	24
					Male	0	16 to 34	44.7	25.1	66.1	34	12
					Male	0	16 to 85	35.7	24.9	48.2	149	47
					Male	0	35 to 44	41	19	67.3	43	16
					Male	0	45 to 54	29.8	11	59.4	40	11
					Male	0	55 to 85	20.3	9.4	38.6	32	8
		Minimally adequate treatment			Both	62.2	16 to 85	29	23.3	36	442	128
					Female	100	16 to 34	20.5	13.5	29.9	106	25
					Female	100	16 to 85	33.4	26.8	40.8	293	96
					Female	100	35 to 44	34.3	20.4	51.6	62	26
					Female	100	45 to 54	48.6	25.8	72	49	20
					Female	100	55 to 85	36.7	24.1	51.5	76	25
					Male	0	16 to 34	23.5	9	48.6	34	7
					Male	0	16 to 85	21.7	12.4	35.2	149	32
					Male	0	35 to 44	26.7	9.1	56.9	43	11
					Male	0	45 to 54	17.6	3.9	53.2	40	8

	12 months	Minimally adequate treatment			Male	0	55 to 85	15.3	4.7	39.8	32	6
Bogner HR, de Vries HF, Maulik PK, Unützer J. Mental health services use: Baltimore epidemiologic catchment area follow-up. Am J Geriatr Psychiatry. 2009; 17(8): 706-15.	6 months	Any mental health service	Maryland	2004 to 2005	Both	Not reported	60 to 99	34.6	19.4	53.8	26	9
Borges G, Medina-Mora ME, Wang PS, Lara C, Berglund P, Walters E. Treatment and adequacy of treatment of mental disorders among respondents to the Mexico National Comorbidity Survey. Am J Psychiatry. 2006; 163(8): 1371-8.	12 months	Any mental health service	Mexico	2001 to 2002	Both	76.4	18 to 65	12.6	7.7	17.5	240	30.24
Briggs R, Tobin K, Kenny RA, Kennelly SP. What is the prevalence of untreated depression and death ideation in older people? Data from the Irish Longitudinal Study on Aging. Int Psychogeriatr. 2018; 30(9): 1393-1401.	12 months	Antidepressants	Ireland	2010 to 2012	Both	64.8	50 to 64	29.8	26	33.9	513	153
					Both	64.8	65 to 74	28	22.3	34.5	207	58
					Both	64.8	75 to 99	25.2	18.3	33.7	119	30
					Female	100	50 to 99	30.5	26.8	34.5	544	166
					Male	0	50 to 99	25.4	20.8	30.7	295	75
Cia AH, Stagnaro JC, Aguilar-Gaxiola S, Sustas S, Serfaty E, Nemirovsky M, Kessler RC, Benjet C. Twelve-month utilization rates and adequacy of treatment for mental health and substance use disorders in Argentina. Braz J Psychiatry. 2019; 41(3): 238-244.	12 months	Any mental health service	Argentina	2015 to 2015	Both	Not reported	18 to 98	29.8	22.5	37	211	63
Duhoux A, Fournier L, Nguyen CT, Roberge P, Beveridge R. Guideline concordance of treatment for depressive disorders in Canada. Soc Psychiatry Psychiatr Epidemiol. 2009; 44(5): 385-92.	12 months	Minimally adequate treatment	Canada	2002 to 2002	Both	63.9	15 to 99	29.1	26.6	31.6	1563	455
Fernández A, Haro JM, Codony M, Vilagut G, Martínez-Alonso M, Autonell J, Salvador-Carulla L, Ayuso-Mateos JL, Fullana MA, Alonso J. Treatment adequacy of anxiety and depressive disorders: primary versus specialised care in Spain. J Affect Disord. 2006; 96(1-2): 9-20.	12 months	Any mental health service	Spain	2001 to 2002	Both	77.3	18 to 98	34.4	28.9	40.4	262	88
Gadalla TM. Comparison of users and non-users of mental health services among depressed women: a national study. Women Health. 2008; 47(1): 1-19.	12 months	Any mental health service	Canada	2002 to 2002	Female	100	15 to 99	42	39.2	44.8	1186	498
Goldney RD, Fisher LJ, Grande ED, Taylor AW, Hawthorne G. Have education and publicity	1 month	Any mental health service	Australia	1998 to 1998	Both	Not reported	15 to 99	17.6	13	23.4	205	36

about depression made a difference? Comparison of prevalence, service use and excess costs in South Australia: 1998 and 2004. Aust N Z J Psychiatry. 2007; 41(1): 38-53.				2004 to 2004	Both	Not reported	15 to 99	19.9	15.3	25.4	241	48
Gureje O, Uwakwe R, Oladeji B, Makanjuola VO, Esan O. Depression in adult Nigerians: results from the Nigerian Survey of Mental Health and Well-being. J Affect Disord. 2010; 120(1-3): 158-64.	12 months	Any mental health service	Nigeria	2002 to 2003	Both	47.9	18 to 100	4	0	8	115	5
Gwynn RC, McQuistion HL, McVeigh KH, Garg RK, Frieden TR, Thorpe LE. Prevalence, diagnosis, and treatment of depression and generalized anxiety disorder in a diverse urban community. Psychiatr Serv. 2008; 59(6): 641-7.	12 months	Any mental health service	New York	2004 to 2004	Both	64.3	20 to 99	31	24.1	38.9	148	6
Hämäläinen J, Isometsä E, Sihvo S, Pirkola S, Kiviruusu O. Use of health services for major depressive and anxiety disorders in Finland. Depress Anxiety. 2008; 25(1): 27-37.	12 months	Antidepressants	Finland	2000 to 2001	Both	68.7	30 to 99	32	23	42.6	84	7
					Both	68.7	30 to 39	28	19.2	39	76	21
					Both	68.7	30 to 99	24	19.4	29.3	288	69
					Both	68.7	40 to 49	22	15	31	101	22
					Both	68.7	50 to 59	20	12.3	30.9	69	14
					Both	68.7	60 to 99	31	19.1	46.1	42	13
					Female	100	30 to 99	27	21.3	33.6	198	53
					Male	0	30 to 99	19	12.3	28.1	93	18
		Any mental health service			Both	68.7	30 to 39	21	13.5	31.1	81	17
					Both	68.7	30 to 99	24.9	20.3	30.1	297	74
					Both	68.7	40 to 49	26.9	19.3	36.2	104	28
					Both	68.7	50 to 59	23.5	15	34.9	68	16
					Both	68.7	60 to 99	9.3	0	19.1	43	4
					Female	100	30 to 99	22.1	16.9	28.2	204	45
					Male	0	30 to 99	21.5	14.4	30.9	93	20
		Minimally adequate treatment		2000 to 2001	Both	69	30 to 99	17.7	13.7	22.5	288	51
Jeuring HW, Comijs HC, Deeg DJH, Stek ML, Huisman M, Beekman ATF. Secular trends in the prevalence of major and subthreshold depression among 55-64-year olds over 20 years. Psychol Med. 2018; 48(11): 1824-1834.	1 months	Antidepressants	Netherlands	1992 to 2013	Both	69.1	55 to 64	26.6	18.7	36.3	94	25

Kazdin AE, Wu CS, Hwang I, Puac-Polanco V, Sampson NA, Al-Hamzawi A, Alonso J, Andrade LH, Benjet C, Caldas-de-Almeida JM, de Girolamo G, de Jonge P, Florescu S, Gureje O, Haro JM, Harris MG, Karam EG, Karam G, Kovess-Masfety V, Lee S, McGrath JJ, Navarro-Mateu F, Nishi D, Oladeji BD, Posada-Villa J, Stein DJ, Üstün TB, Vigo DV, Zarkov Z, Zaslavsky AM, Kessler RC, WHO World Mental Health Survey collaborators. Antidepressant use in low- middle- and high-income countries: a World Mental Health Surveys report. Psychol Med. 2023; 53(4): 1583-1591.	12 months	Antidepressents	Argentina	2015 to 2015	Both	Not reported	18 to 98	5.8	1.3	10.3	Not reported	Not reported
			Belgium	2001 to 2002	Both	Not reported	18 to 95	41.1	27.4	54.8	Not reported	Not reported
			Bulgaria	2003 to 2007	Both	Not reported	18 to 98	5.5	1	10	Not reported	Not reported
			Colombia	2003 to 2003	Both	Not reported	18 to 65	7.3	2	12.6	Not reported	Not reported
			Colombia	2011 to 2012	Both	Not reported	19 to 65	14.2	8.1	20.3	Not reported	Not reported
			France	2001 to 2002	Both	Not reported	18 to 97	30.8	21.6	40	Not reported	Not reported
			Germany	2002 to 2003	Both	Not reported	19 to 95	33.9	23.9	43.9	Not reported	Not reported
			Iraq	2006 to 2007	Both	Not reported	18 to 96	4	0	8.9	Not reported	Not reported
			Israel	2002 to 2004	Both	Not reported	21 to 98	13.6	9.5	17.7	Not reported	Not reported
			Italy	2001 to 2002	Both	Not reported	18 to 100	17.2	10.9	23.5	Not reported	Not reported
			Japan	2002 to 2003	Both	Not reported	20 to 98	12.1	3.5	20.7	Not reported	Not reported
			Lebanon	2002 to 2003	Both	Not reported	18 to 94	6	0.9	11.1	Not reported	Not reported
			Mexico	2001 to 2002	Both	76.4	18 to 65	5.8	2.1	9.5	Not reported	Not reported
			Netherlands	2002 to 2003	Both	Not reported	18 to 95	29.9	17.6	42.2	Not reported	Not reported
			Nigeria	2002 to 2003	Both	47.9	18 to 100	0	0	0	Not reported	Not reported
			Peru	2004 to 2005	Both	Not reported	18 to 65	5	0.3	9.7	Not reported	Not reported
			Portugal	2008 to 2009	Both	Not reported	18 to 81	32.8	26.7	38.9	Not reported	Not reported
			Romania	2005 to 2006	Both	Not reported	18 to 96	11.1	0.3	21.9	Not reported	Not reported
			São Paulo	2005 to 2007	Both	Not reported	18 to 93	16.6	12.3	20.9	Not reported	Not reported
			Spain	2001 to 2002	Both	77.3	18 to 98	28.5	22	35	Not reported	Not reported
			Spain	2010 to 2012	Both	Not reported	18 to 96	23.3	11	35.6	Not reported	Not reported
			United States of America	2001 to 2003	Both	66.3	18 to 99	29.1	25	33.2	Not reported	Not reported

Le LK, Shih S, Richards-Jones S, Chatterton ML, Engel L, Stevenson C, Lawrence D, Pepin G, Mihalopoulos C. The cost of Medicare-funded medical and pharmaceutical services for mental disorders in children and adolescents in Australia. PLoS One. 2021; 16(4): e0249902.	12 months	Any mental health service	Australia	2013 to 2014	Both	55.4	4 to 17	14.9	5.6	23	Not reported	Not reported
Lee S, Tsang A, Huang Y-Q, He Y-L, Liu ZR, Zhang M-Y, Shen Y-C, Kessler RC. The epidemiology of depression in metropolitan China. Psychol Med. 2009; 39(5): 735-47.	12 months	Any mental health service	Beijing (population-weighted)	2001 to 2002	Both	48.3	18 to 99	5.2	0	13	45	2
			Shanghai (population-weighted)	2001 to 2002	Both	48.3	18 to 99	5.2	0	13.1	44	2
Leles da Costa Dias F, Teixeira AL, Cerqueira Guimarães H, Borges Santos AP, Rios Fonseca Ritter S, Barbosa Machado JC, Tonidandel Barbosa M, Caramelli P. Prevalence of late-life depression and its correlates in a community-dwelling low-educated population aged 75+ years: The Pietà study. J Affect Disord. 2019; 242: 173-179.	1 months	Antidepressants	Minas Gerais	2007 to 2007	Both	75.7	75 to 99	15.7	9	26	70	11
Lu J, Xu X, Huang Y, Li T, Ma C, Xu G, Yin H, Xu X, Ma Y, Wang L, Huang Z, Yan Y, Wang B, Xiao S, Zhou L, Li L, Zhang Y, Chen H, Zhang T, Yan J, Ding H, Yu Y, Kou C, Shen Z, Jiang L, Wang Z, Sun X, Xu Y, He Y, Guo W, Jiang L, Li S, Pan W, Wu Y, Li G, Jia F, Shi J, Shen Z, Zhang N. Prevalence of depressive disorders and treatment in China: a cross-sectional epidemiological study. Lancet Psychiatry. 2021; 8(11): 981-990.	12 months	Any mental health service	China	2013 to 2015	Both	66.6	18 to 100	4.7	2.2	7.2	655	33
		Minimally adequate treatment			Both	66.6	18 to 100	0.8	0.2	1.5	655	11
Ministry of Health and Welfare (South Korea). South Korea National Mental Health Survey 2021.	12 months	Any mental health service	Republic of Korea	2021 to 2021	Both	70.5	18 to 79	28.2	17.8	38.6	94	26
Naganuma Y, Tachimori H, Kawakami N, Takeshima T, Ono Y, Uda H, Hata Y, Nakane Y, Nakane H, Iwata N, Furukawa TA, Kikkawa T. Twelve-month use of mental health services in four areas in Japan: findings from the World Mental Health Japan Survey 2002-2003. Psychiatry Clin Neurosci. 2006; 60(2): 240-8.	12 months	Any mental health service	Japan	2002 to 2003	Both	Not reported	20 to 98	25.2	12.5	37.9	42	11
Oakley Browne MA, Wells JE, McGee MA, New Zealand Mental Health Survey Research Team. Twelve-month and lifetime health service use in Te Rau Hinengaro: The New Zealand Mental Health Survey. Aust N Z J Psychiatry. 2006; 40(10): 855–64.	12 months	Any mental health service	New Zealand	2003 to 2004	Both	70.5	16 to 99	26.2	22.3	30.4	741	194

Rivera M, Porras-Segovia A, Rovira P, Molina E, Gutiérrez B, Cervilla J. Associations of major depressive disorder with chronic physical conditions, obesity and medication use: Results from the PISMA-ep study. Eur Psychiatry. 2019; 60: 20-27.	1 months	Antidepressants	Spain	2013 to 2014	Both	68.5	18 to 75	21.4	17.1	26.4	295	63
Seedat S, Williams DR, Herman AA, Moomal H, Williams SL, Jackson PB, Myer L, Stein DJ. Mental health service use among South Africans for mood, anxiety and substance use disorders. S Afr Med J. 2009; 99(5 Pt 2): 346-52.	12 months	Any mental health service	South Africa	2002 to 2004	Female	100	18 to 99	9	1.7	16.3	170	15.3
				2002 to 2004	Male	0	18 to 99	1.2	0	3.6	53	1
Sigström R, Waern M, Gudmundsson P, Skoog I, Östling S. Depressive spectrum states in a population-based cohort of 70-year olds followed over 9 years. Int J Geriatr Psychiatry. 2018; 33(8): 1028-1037.	1 month	Antidepressants	Sweden	2000 to 2001	Both	75	70 to 70	25	3.7	46.3	20	5
Sonnenberg CM, Deeg DJ, Comijs HC, van Tilburg W, Beekman AT. Trends in antidepressant use in the older population: results from the LASA-study over a period of 10 years. J Affect Disord. 2008; 111(2-3): 299-305.	1 month	Antidepressants	Netherlands	1992 to 1993	Both	Not reported	65 to 85	15	7.1	29.1	40	6
				1995 to 1996	Both	Not reported	68 to 88	5.3	0	14	38	2
				1998 to 1999	Both	Not reported	71 to 91	21.4	11.7	35.9	42	9
				2001 to 2004	Both	Not reported	74 to 94	30.4	15.6	50.9	23	7
Ten Have M, de Graaf R, van Dorsselaer S, Tuithof M, Kleinjan M, Penninx BWJH. Recurrence and chronicity of major depressive disorder and their risk indicators in a population cohort. Acta Psychiatr Scand. 2018; 137(6): 503-515.	12 months	Any mental health service	Netherlands	2007 to 2009	Both	69.8	18 to 64	39.8	33.8	46.1	242	95
Thornicroft G, Chatterji S, Evans-Lacko S, Gruber M, Sampson N, Aguilar-Gaxiola S, Al-Hamzawi A, Alonso J, Andrade L, Borges G, Bruffaerts R, Bunting B, de Almeida JM, Florescu S, de Girolamo G, Gureje O, Haro JM, He Y, Hinkov H, Karam E, Kawakami N, Lee S, Navarro-Mateu F, Piazza M, Posada-Villa J, de Galvis YT, Kessler RC. Undertreatment of people with major depressive disorder in 21 countries. Br J Psychiatry. 2017; 210(2): 119-124.	12 months	Minimally adequate treatment	Argentina	2015 to 2015	Both	Not reported	18 to 98	17.9	12.6	23.2	170	30
			Belgium	2001 to 2002	Both	Not reported	18 to 95	29.5	17.7	41.3	105	31
			Bulgaria	2003 to 2007	Both	Not reported	18 to 98	6.7	2.2	11.2	145	10
			Colombia	2003 to 2003	Both	Not reported	18 to 65	5	0.3	9.7	241	12
				2011 to 2012	Both	Not reported	19 to 65	9	3.7	14.3	151	14
			France	2001 to 2002	Both	Not reported	18 to 97	23	13.4	32.6	158	36
			Germany	2002 to 2003	Both	Not reported	19 to 95	31.6	23.4	39.8	109	34

			Iraq	2006 to 2007	Both	Not reported	18 to 96	2.5	0	7.2	182	5
			Israel	2002 to 2004	Both	Not reported	21 to 98	15.8	11.5	20.1	280	44
			Italy	2001 to 2002	Both	Not reported	18 to 100	16.7	9.4	24	119	20
			Japan	2002 to 2003	Both	Not reported	20 to 98	22.2	12.4	32	81	18
			Lebanon	2002 to 2003	Both	Not reported	18 to 94	7	3.7	10.3	126	92
			Mexico	2001 to 2002	Both	76.4	18 to 65	6.4	3.5	9.3	231	15
			Netherlands	2002 to 2003	Both	Not reported	18 to 95	33.1	23.1	43.1	125	41
			Nigeria	2002 to 2003	Both	47.9	18 to 100	0	0	2.7	72	0
			Peru	2004 to 2005	Both	Not reported	18 to 65	0.9	0	2.7	99	1
			Portugal	2008 to 2009	Both	Not reported	18 to 81	18.8	13.5	24.1	290	55
			Romania	2005 to 2006	Both	Not reported	18 to 96	13.5	0	28.2	40	5
			São Paulo	2005 to 2007	Both	Not reported	18 to 93	14.9	11	18.8	489	73
			Spain	2001 to 2002	Both	77.3	18 to 98	27.2	20.9	33.5	231	63
				2010 to 2012	Both	Not reported	18 to 96	18.8	11.9	25.7	154	29
			United States of America	2001 to 2003	Both	66.3	18 to 99	26.6	22.9	30.3	646	172
Trimbos Institute (Netherlands). Netherlands Mental Health Survey and Incidence Study 2019-2022.	12 months	Any mental health service	Netherlands	2019 to 2022	Both	61.7	18 to 75	33.5	29	38.1	526	176
Wang PS, Lane M, Olfson M, Pincus HA, Wells KB, Kessler RC. Twelve-month use of mental health services in the United States: results from the National Comorbidity Survey Replication. Arch Gen Psychiatry. 2005; 62(6): 629-40.	12 months	Any mental health service	United States of America	2001 to 2003	Both	66.3	18 to 99	32.9	29.8	36	623	205

Table S3: Candidate models for the estimation of bias corrections for non-reference data

Step	Covariate	Coefficient (95% UI)	*p*	AIC	BIC
Model 1	Antidepressants	0.222 (-0.038 to 0.482)	0.09447	54.46	64.85
	Any mental health service utilisation	0.070 (-0.163 to 0.304)	0.5564		
	Interaction: HAQI x Any mental health service utilisation	-0.012 (-0.023 to -0.001)	0.03602		
	Interaction: HAQI x Antidepressants[1]	0.002 (-0.008 to 0.012)	0.7258		
Model 2[1]	Antidepressants	0.179 (0.072 to 0.287)	0.001042	52.59	60.90
	Any mental health service utilisation[1]	0.062 (-0.167 to 0.292)	0.5947		
	Interaction: HAQI x Any mental health service utilisation	-0.012 (-0.024 to -0.001)	0.02872		

1 = Final model chosen based on Akaike information criterion. AIC = Akaike information criterion. BIC = Bayesian information criterion.

HAQI = Healthcare access quality index.

Table S4: Candidate scale transformations for the HAQI in the estimation of bias corrections for non-reference data

HAQI Scale	Step	Covariate	Coefficient (95% UI)	*p*	AIC	BIC
Square-root	Model 1	Antidepressants	0.214 (-0.02 to 0.448)	0.07283	54.17	64.55
		Any mental health service utilisation	0.092 (-0.118 to 0.302)	0.3918		
		Interaction: HAQI x Any mental health service utilisation	-0.206 (-0.393 to -0.019)	0.03092		
		Interaction: HAQI x Antidepressants	0.028 (-0.136 to 0.192)	0.7380		
	Model 2	Antidepressants	0.178 (0.071 to 0.286)	0.001094	52.28	60.59
		Any mental health service utilisation	0.085 (-0.121 to 0.291)	0.4203		
		Interaction: HAQI x Any mental health service utilisation	-0.212 (-0.396 to -0.028)	0.02406		
Cubed-root	Model 1	Antidepressants	0.211 (-0.014 to 0.437)	0.06647	54.08	64.46
		Any mental health service utilisation	0.099 (-0.104 to 0.302)	0.3386		
		Interaction: HAQI x Any mental health service utilisation	-0.628 (-1.194 to -0.063)	0.02951		
		Interaction: HAQI x Antidepressants	0.083 (-0.411 to 0.576)	0.7424		
	Model 2	Antidepressants	0.178 (0.071 to 0.285)	0.001113	52.18	60.49
		Any mental health service utilisation	0.092 (-0.106 to 0.291)	0.3632		
		Interaction: HAQI x Any mental health service utilisation	-0.646 (-1.201 to -0.09)	0.02278		
Squared	Model 1	Antidepressants	0.239 (-0.085 to 0.564)	0.1486	55.13	65.52
		Any mental health service utilisation	0.029 (-0.259 to 0.316)	0.8446		
		Interaction: HAQI x Any mental health service utilisation	> -0.001 (> -0.001 to < 0.000)	0.05128		
		Interaction: HAQI x Antidepressants	< 0.001 (> 0.000 to < 0.001)	0.7082		
	Model 2	Antidepressants	0.181 (0.073 to 0.288)	0.000972	53.27	61.581
		Any mental health service utilisation	0.02 (-0.264 to 0.303)	0.8910		
		Interaction: HAQI x Any mental health service utilisation	> -0.001 (> -0.001 to < 0.000)	0.04286		
Cubed	Model 1	Antidepressants	0.259 (-0.15 to 0.667)	0.2144	55.84	66.23
		Any mental health service utilisation	-0.006 (-0.356 to 0.343)	0.9718		
		Interaction: HAQI x Any mental health service utilisation	> -0.001 (> -0.001 to < 0.000)	0.07575		
		Interaction: HAQI x Antidepressants	< 0.001 (> 0.000 to < 0.001)	0.7017		
	Model 2	Antidepressants	0.182 (0.074 to 0.29)	0.00095	53.99	62.30
		Any mental health service utilisation	-0.016 (-0.362 to 0.33)	0.9285		
		Interaction: HAQI x Any mental health service utilisation	> -0.001 (> -0.001 to < 0.000)	0.06604		

HAQI = Healthcare access quality index.

Table S5: Proportion of persons with major depressive disorder receiving minimally adequate treatment by sex and location for 2000 and 2021 (95% UIs).

Location	2000			2021		
	Both	Females	Males	Both	Females	Males
**Global**	**9·2 (7·4–11·7)**	**10·3 (8·3–13·1)**	**7·4 (5·9–9·4)**	**9·1 (7·2–11·6)**	**10·2 (8·2–13·1)**	**7·2 (5·7–9·3)**
**Central Europe, Eastern Europe, and Central Asia**	**11·3 (9·1–14·3)**	**12·5 (10·1–15·9)**	**9·0 (7·2–11·6)**	**12·6 (10·1–16·1)**	**13·9 (11·1–17·8)**	**10·3 (8·2–13·2)**
**Central Asia**	**9·6 (7·0–13·3)**	**10·7 (7·8–14·7)**	**7·6 (5·3–10·8)**	**10·8 (7·8–15·2)**	**12·0 (8·8–16·6)**	**8·7 (6·1–12·6)**
Armenia	10·8 (7·9–15·0)	11·8 (8·6–16·4)	8·4 (6·0–12·1)	12·0 (8·7–16·6)	13·2 (9·6–18·6)	9·4 (6·9–13·0)
Azerbaijan	9·6 (6·8–13·6)	10·6 (7·5–14·8)	7·4 (5·1–10·7)	11·0 (7·9–15·9)	12·1 (8·6–17·3)	8·6 (6·0–12·3)
Georgia	11·0 (8·0–15·3)	12·1 (8·8–16·6)	8·8 (6·4–12·2)	11·4 (8·3–16·0)	12·6 (9·1–17·6)	9·3 (6·8–12·8)
Kazakhstan	9·7 (7·0–13·5)	10·8 (7·9–15·1)	7·6 (5·3–11·1)	11·4 (8·2–15·9)	12·6 (9·2–17·4)	9·3 (6·4–13·4)
Kyrgyzstan	9·4 (6·7–13·1)	10·4 (7·6–14·5)	7·5 (5·1–10·9)	10·6 (7·5–15·0)	11·8 (8·3–16·6)	8·6 (5·9–12·5)
Mongolia	8·7 (6·0–12·8)	9·8 (6·8–14·3)	7·0 (4·7–10·4)	10·6 (7·4–15·1)	11·8 (8·2–16·6)	8·6 (5·9–12·7)
Tajikistan	8·8 (6·2–12·6)	9·7 (6·9–13·8)	6·9 (4·6–10·2)	9·8 (7·0–14·1)	10·8 (7·7–15·5)	7·7 (5·3–11·3)
Turkmenistan	9·0 (6·4–12·8)	10·1 (7·2–14·3)	7·1 (4·8–10·4)	9·9 (7·0–14·1)	11·0 (7·8–15·4)	7·9 (5·4–11·4)
Uzbekistan	9·4 (6·6–13·3)	10·4 (7·4–14·8)	7·5 (5·1–10·8)	10·6 (7·6–15·0)	11·8 (8·6–16·5)	8·6 (6·0–12·5)
**Central Europe**	**12·4 (9·5–17·0)**	**13·5 (10·2–18·6)**	**9·8 (7·5–13·0)**	**13·6 (10·4–18·2)**	**14·7 (11·1–19·7)**	**10·9 (8·4–14·4)**
Albania	11·0 (7·9–15·5)	11·9 (8·5–16·6)	8·4 (6·0–12·0)	12·6 (9·2–17·6)	13·5 (9·9–18·9)	9·7 (7·0–13·6)
Bosnia and Herzegovina	11·7 (8·6–16·6)	12·7 (9·3–18·0)	8·8 (6·2–12·1)	12·8 (9·4–17·9)	13·7 (10·0–19·2)	10·0 (7·3–13·8)
Bulgaria	8·7 (6·4–11·6)	9·3 (6·9–12·6)	6·7 (4·9–9·0)	9·5 (6·9–13·0)	10·1 (7·4–13·8)	7·3 (5·4–10·3)
Croatia	12·8 (9·4–18·1)	13·9 (10·0–19·7)	10·0 (7·2–13·9)	13·8 (10·1–19·1)	14·9 (10·8–20·7)	11·0 (8·1–15·2)
Czechia	13·1 (9·6–18·8)	14·1 (10·3–20·0)	10·2 (7·3–14·6)	14·1 (10·2–19·6)	15·1 (10·8–21·0)	11·2 (8·2–15·8)
Hungary	12·5 (9·2–17·7)	13·5 (9·8–19·3)	10·0 (7·0–14·0)	13·4 (9·8–18·7)	14·3 (10·4–20·0)	10·7 (7·8–15·0)
Montenegro	12·4 (9·0–17·1)	13·4 (9·7–18·4)	9·5 (6·9–13·3)	13·5 (9·8–18·8)	14·5 (10·5–20·3)	10·4 (7·5–14·7)
North Macedonia	11·6 (8·5–16·5)	12·5 (9·0–17·8)	8·9 (6·4–12·5)	12·8 (9·3–17·8)	13·7 (9·9–19·2)	10·0 (7·3–14·1)
Poland	11·5 (9·6–14·1)	12·9 (10·9–15·6)	9·4 (7·8–11·6)	12·6 (10·6–15·2)	14·0 (11·8–16·9)	10·6 (8·9–12·9)
Romania	15·4 (11·4–21·5)	16·7 (12·2–23·4)	12·1 (8·6–16·7)	17·0 (12·4–24·0)	18·3 (13·2–25·6)	13·7 (10·1–19·1)
Serbia	11·7 (8·6–16·4)	12·6 (9·2–17·8)	9·1 (6·5–12·7)	12·8 (9·4–17·7)	13·7 (10·1–19·2)	10·2 (7·5–14·0)
Slovakia	12·1 (8·9–17·1)	13·0 (9·4–18·5)	9·4 (6·7–13·2)	13·4 (9·9–19·0)	14·3 (10·4–19·9)	10·6 (7·6–14·9)
Slovenia	13·2 (9·8–18·6)	14·2 (10·4–20·0)	10·4 (7·4–14·6)	15·0 (10·9–20·7)	15·9 (11·6–22·0)	12·0 (8·8–16·7)

**Eastern Europe**	**11·2 (9·3–13·9)**	**12·5 (10·4–15·4)**	**9·1 (7·5–11·4)**	**12·8 (10·6–15·8)**	**14·2 (11·8–17·5)**	**10·5 (8·7–13·0)**
Belarus	11·8 (8·7–16·7)	13·0 (9·6–18·4)	9·6 (6·8–13·7)	13·5 (9·8–19·1)	14·9 (10·9–21·2)	10·9 (7·9–15·2)
Estonia	12·0 (8·7–16·8)	13·1 (9·5–18·3)	9·7 (6·9–13·8)	13·7 (10·0–19·0)	14·9 (11·0–20·5)	11·1 (8·2–15·6)
Latvia	11·8 (8·6–16·6)	13·0 (9·5–18·2)	9·6 (6·9–13·6)	12·9 (9·4–17·8)	14·2 (10·3–19·5)	10·5 (7·6–14·8)
Lithuania	12·2 (8·9–17·2)	13·4 (9·8–19·0)	9·8 (7·0–14·1)	12·9 (9·5–18·2)	14·2 (10·4–20·1)	10·5 (7·7–14·6)
Republic of Moldova	11·1 (8·1–15·5)	12·3 (8·9–17·1)	9·0 (6·4–12·5)	12·3 (8·9–17·2)	13·5 (9·8–18·9)	10·1 (7·3–14·2)
Russian Federation	10·9 (9·1–13·2)	12·2 (10·3–14·7)	8·9 (7·4–11·0)	12·8 (10·8–15·6)	14·2 (12·0–17·2)	10·6 (8·9–13·0)
Ukraine	11·8 (9·7–14·3)	12·9 (10·7–15·7)	9·3 (7·7–11·5)	12·6 (10·6–15·3)	13·9 (11·6–16·8)	10·2 (8·5–12·5)
**High-income**	**25·7 (20·7–32·7)**	**27·4 (22·3–34·8)**	**22·4 (17·7–28·6)**	**27·0 (21·7–34·4)**	**29·0 (23·4–36·8)**	**23·4 (18·7–30·3)**
**Australasia**	**25·4 (21·1–30·8)**	**27·4 (22·9–32·8)**	**22·3 (17·6–27·6)**	**29·2 (21·4–40·8)**	**31·9 (23·2–44·6)**	**25·2 (18·2–35·8)**
Australia	25·8 (21·4–31·2)	27·8 (23·2–33·5)	22·6 (17·7–28·2)	30·1 (21·6–42·6)	33·0 (23·5–46·7)	25·9 (18·4–37·3)
New Zealand	22·8 (18·7–28·0)	24·5 (20·1–30·2)	20·0 (16·3–24·4)	22·8 (19·0–27·7)	24·6 (20·5–29·8)	20·1 (16·8–24·3)
**High-income Asia Pacific**	**24·7 (20·2–31·1)**	**27·0 (22·2–33·8)**	**20·8 (16·9–26·4)**	**26·4 (21·9–32·4)**	**29·1 (24·3–35·5)**	**22·0 (18·2–27·4)**
Brunei Darussalam	19·3 (12·7–28·6)	21·2 (13·8–31·8)	16·0 (10·6–23·8)	21·2 (14·5–31·0)	23·3 (16·1–34·3)	17·5 (11·7–25·5)
Japan	24·3 (20·5–29·2)	26·7 (22·6–31·9)	20·4 (17·1–24·7)	25·0 (21·4–29·6)	27·6 (23·5–32·5)	20·9 (17·8–25·1)
Republic of Korea	26·3 (18·5–37·5)	28·4 (20·1–40·1)	22·5 (15·2–32·5)	30·0 (23·1–39·8)	33·1 (25·5–43·8)	24·8 (18·6–33·4)
Singapore	24·8 (16·8–36·6)	26·6 (18·2–39·1)	21·5 (14·2–31·9)	28·2 (20·4–40·6)	31·0 (22·7–44·2)	24·0 (17·1–35·2)
**High-income North America**	**26·7 (22·0–32·6)**	**28·3 (23·3–34·5)**	**23·8 (19·3–29·2)**	**27·8 (22·8–34·3)**	**29·6 (24·2–36·6)**	**24·3 (20·1–29·9)**
Canada	33·3 (24·6–45·3)	35·5 (26·4–47·0)	29·1 (20·8–42·1)	36·2 (26·1–50·0)	39·0 (28·2–54·7)	31·0 (22·0–45·0)
Greenland	25·8 (16·8–37·9)	27·3 (17·9–39·8)	23·3 (15·2–34·6)	26·9 (18·4–38·8)	28·9 (19·6–41·7)	23·7 (16·3–34·6)
United States of America	26·2 (21·6–31·7)	27·8 (22·8–33·7)	23·4 (19·0–28·4)	27·1 (22·4–33·2)	28·9 (23·8–35·3)	23·8 (19·7–28·9)
**Southern Latin America**	**21·4 (15·1–30·7)**	**23·0 (16·2–32·8)**	**18·0 (12·7–26·0)**	**22·4 (16·0–31·6)**	**24·1 (17·4–33·9)**	**19·1 (13·5–26·9)**
Argentina	20·7 (14·7–30·0)	22·3 (15·7–32·3)	17·4 (12·5–25·0)	20·8 (15·0–29·6)	22·3 (16·1–31·6)	18·1 (12·7–25·8)
Chile	22·1 (15·3–31·7)	23·6 (16·2–33·8)	18·8 (12·8–27·7)	24·6 (17·6–35·3)	26·3 (18·7–37·6)	20·8 (14·6–29·9)
Uruguay	22·8 (16·6–32·2)	24·7 (18·1–34·5)	18·6 (13·1–26·2)	23·9 (17·1–34·2)	25·6 (18·4–36·8)	19·9 (14·0–28·7)
**Western Europe**	**25·5 (20·0–33·4)**	**27·3 (21·4–35·4)**	**22·0 (17·0–29·6)**	**26·9 (20·9–35·7)**	**28·9 (22·4–38·1)**	**23·2 (18·0–31·5)**
Andorra	27·2 (19·1–39·3)	29·2 (20·5–42·0)	23·8 (16·6–34·7)	27·7 (19·7–40·0)	30·0 (21·4–43·1)	23·9 (16·9–34·8)
Austria	25·8 (18·6–36·4)	27·4 (19·8–38·3)	22·1 (15·3–32·0)	26·7 (19·5–37·5)	28·5 (20·9–39·5)	22·8 (16·4–32·9)
Belgium	32·4 (24·0–44·1)	34·8 (26·2–47·4)	28·0 (20·1–38·7)	34·4 (25·2–47·6)	37·0 (27·0–51·0)	29·7 (21·3–42·2)

Cyprus	22·0 (15·6–31·9)	23·6 (16·8–34·2)	18·6 (13·1–27·1)	26·8 (19·0–38·7)	28·7 (20·3–41·3)	22·4 (16·0–31·9)
Denmark	24·8 (17·8–35·2)	26·4 (19·3–37·4)	21·2 (15·0–30·4)	26·0 (19·1–36·5)	27·8 (20·4–38·7)	22·2 (16·3–31·9)
England	24·5 (20·8–29·4)	26·6 (22·6–31·9)	21·4 (17·9–26·0)	25·8 (21·7–31·2)	28·1 (23·7–33·9)	22·4 (18·8–27·3)
Finland	17·9 (14·8–22·2)	19·2 (15·8–23·7)	15·8 (12·9–20·0)	20·6 (15·2–29·0)	22·0 (16·3–31·1)	18·3 (13·2–26·1)
France	26·2 (19·0–37·0)	28·0 (20·4–39·3)	22·3 (15·9–31·7)	29·0 (21·3–40·7)	31·2 (23·0–43·7)	24·5 (17·7–35·3)
Germany	32·9 (24·7–43·7)	35·3 (26·6–46·7)	28·8 (21·1–39·5)	35·0 (26·0–48·0)	37·7 (28·0–51·5)	30·5 (22·7–43·0)
Greece	25·6 (18·8–36·5)	27·6 (20·3–39·1)	22·0 (15·9–31·1)	25·6 (18·8–35·8)	27·7 (20·4–38·9)	21·7 (16·1–30·1)
Iceland	25·9 (18·2–37·1)	27·6 (19·3–39·4)	22·5 (15·7–32·7)	26·6 (19·3–37·1)	28·4 (20·4–39·5)	23·2 (16·8–33·3)
Ireland	26·7 (19·0–38·7)	27·7 (19·9–40·1)	25·0 (17·5–36·6)	30·1 (21·3–42·9)	31·3 (22·2–44·6)	28·0 (19·8–39·8)
Israel	18·0 (12·9–25·3)	19·6 (14·1–27·7)	14·8 (10·6–21·0)	20·0 (14·6–28·3)	21·7 (16·0–30·7)	16·7 (11·9–23·9)
Italy	19·9 (17·0–23·9)	21·5 (18·3–25·7)	16·4 (13·8–19·7)	19·5 (16·6–23·4)	21·2 (18·0–25·3)	15·9 (13·5–19·2)
Luxembourg	25·6 (18·3–37·0)	27·4 (19·7–39·3)	22·2 (15·3–32·6)	26·9 (19·3–38·2)	28·9 (20·9–40·7)	23·2 (16·4–33·2)
Malta	23·6 (17·2–34·0)	25·3 (18·7–36·4)	20·2 (14·4–29·1)	26·3 (19·6–36·4)	28·2 (21·0–39·0)	22·2 (16·4–31·4)
Monaco	26·0 (19·3–36·8)	28·1 (20·8–39·8)	22·2 (16·2–31·5)	26·2 (19·4–36·7)	28·3 (21·0–39·5)	22·2 (16·4–31·5)
Netherlands	31·2 (24·0–39·2)	33·1 (25·8–41·6)	27·5 (20·5–35·4)	30·3 (23·8–39·7)	32·7 (25·7–42·6)	26·0 (20·4–34·5)
Northern Ireland	24·5 (17·4–34·9)	26·2 (18·8–37·5)	21·1 (14·9–30·6)	26·1 (18·7–37·4)	28·1 (20·2–39·9)	22·5 (16·0–32·4)
Norway	25·5 (21·3–31·0)	27·4 (23·0–33·4)	22·3 (18·7–27·2)	26·7 (22·5–32·3)	29·0 (24·3–35·0)	23·3 (19·6–28·2)
Portugal	19·7 (14·4–27·6)	20·8 (15·2–29·3)	15·8 (11·2–22·5)	21·8 (16·1–30·5)	23·4 (17·3–32·6)	17·6 (13·0–25·1)
San Marino	27·5 (20·1–39·7)	29·5 (21·4–42·7)	23·7 (16·9–33·8)	27·3 (20·0–38·7)	29·5 (21·5–42·0)	23·0 (16·9–32·6)
Scotland	23·7 (17·3–34·1)	25·6 (19·0–35·8)	20·9 (14·9–30·8)	25·7 (18·6–36·8)	27·9 (20·2–39·5)	22·4 (15·8–32·5)
Spain	23·3 (17·4–31·7)	25·3 (19·0–34·2)	19·2 (14·0–26·5)	23·8 (17·9–33·3)	25·6 (19·2–35·6)	19·4 (14·5–26·9)
Sweden	27·2 (19·9–38·2)	28·8 (21·0–40·6)	23·3 (17·0–33·4)	27·7 (20·5–39·3)	29·1 (21·5–41·2)	24·5 (17·9–35·0)
Switzerland	26·6 (18·9–37·9)	28·6 (20·6–40·4)	22·9 (15·9–33·3)	27·9 (20·6–38·7)	30·2 (22·3–42·0)	23·7 (17·3–33·0)
Wales	24·2 (17·7–34·4)	26·1 (19·2–36·5)	20·9 (14·9–30·3)	25·0 (18·3–35·0)	26·9 (19·7–37·5)	22·0 (16·0–31·4)
**Latin America and Caribbean**	**10·1 (8·2–12·8)**	**11·1 (9·0–14·0)**	**8·1 (6·5–10·3)**	**10·8 (8·8–13·6)**	**11·9 (9·6–15·0)**	**8·4 (6·8–10·6)**
**Andean Latin America**	**1·8 (1·2–2·5)**	**1·9 (1·3–2·7)**	**1·5 (1·0–2·2)**	**2·0 (1·4–2·9)**	**2·2 (1·5–3·2)**	**1·7 (1·2–2·5)**
Bolivia (Plurinational State of)	1·7 (1·1–2·4)	1·8 (1·2–2·6)	1·4 (0·9–2·0)	1·9 (1·3–2·8)	2·1 (1·4–3·1)	1·6 (1·1–2·4)
Ecuador	1·8 (1·2–2·6)	1·9 (1·3–2·7)	1·6 (1·1–2·3)	2·0 (1·4–2·9)	2·2 (1·5–3·1)	1·8 (1·2–2·5)
Peru	1·8 (1·2–2·5)	1·9 (1·3–2·7)	1·5 (1·1–2·1)	2·1 (1·5–3·1)	2·3 (1·6–3·4)	1·8 (1·2–2·6)

**Caribbean**	**6·5 (4·6–9·2)**	**7·0 (5·0–10·1)**	**5·4 (3·9–7·7)**	**6·6 (4·8–9·4)**	**7·2 (5·2–10·3)**	**5·6 (4·0–7·9)**
Antigua and Barbuda	6·8 (4·8–9·8)	7·3 (5·1–10·6)	5·5 (3·8–7·7)	7·2 (5·1–10·4)	7·8 (5·5–11·3)	5·8 (4·1–8·3)
Bahamas	6·5 (4·4–9·4)	7·0 (4·8–10·3)	5·3 (3·7–7·7)	6·8 (4·7–9·6)	7·3 (5·1–10·3)	5·5 (3·8–7·9)
Barbados	7·1 (5·1–10·0)	7·7 (5·5–10·9)	5·8 (4·2–8·1)	7·3 (5·4–10·2)	8·0 (5·8–11·3)	6·0 (4·4–8·4)
Belize	5·5 (3·8–8·1)	6·0 (4·1–8·9)	4·8 (3·3–7·0)	6·2 (4·3–9·0)	6·7 (4·6–9·9)	5·2 (3·6–7·6)
Bermuda	8·1 (5·9–11·3)	8·8 (6·4–12·1)	6·8 (4·9–9·8)	8·6 (6·2–12·2)	9·4 (6·8–13·2)	7·1 (5·1–10·1)
Cuba	7·4 (5·3–10·5)	8·0 (5·7–11·5)	6·1 (4·4–8·6)	7·7 (5·6–10·9)	8·4 (6·1–11·9)	6·4 (4·6–9·1)
Dominica	6·2 (4·5–8·7)	6·7 (4·9–9·5)	5·1 (3·6–7·5)	6·3 (4·5–9·0)	6·9 (4·9–9·8)	5·2 (3·8–7·5)
Dominican Republic	6·0 (4·3–8·8)	6·5 (4·6–9·5)	5·0 (3·6–7·2)	6·3 (4·6–9·0)	6·8 (5·0–9·8)	5·3 (3·8–7·6)
Grenada	5·9 (4·2–8·6)	6·5 (4·6–9·3)	5·0 (3·5–7·2)	6·4 (4·6–9·2)	7·0 (5·1–10·1)	5·3 (3·8–7·8)
Guyana	5·5 (3·7–8·1)	5·9 (3·9–8·7)	4·7 (3·2–7·0)	5·6 (3·9–8·2)	6·2 (4·3–8·9)	4·8 (3·3–7·0)
Haiti	4·9 (3·3–7·0)	5·2 (3·5–7·6)	4·1 (2·8–6·0)	5·3 (3·6–7·8)	5·7 (3·8–8·5)	4·4 (3·0–6·4)
Jamaica	6·4 (4·5–9·2)	6·9 (4·9–10·1)	5·2 (3·7–7·3)	6·7 (4·8–9·7)	7·3 (5·2–10·5)	5·5 (3·9–7·7)
Puerto Rico	7·0 (5·1–9·8)	7·8 (5·6–10·9)	6·0 (4·3–8·5)	7·9 (5·8–11·0)	8·7 (6·3–12·1)	6·6 (4·8–9·2)
Saint Kitts and Nevis	6·3 (4·4–9·0)	6·8 (4·8–9·8)	5·3 (3·8–7·5)	6·8 (4·8–9·9)	7·4 (5·2–10·8)	5·7 (3·9–8·2)
Saint Lucia	6·1 (4·3–8·7)	6·6 (4·7–9·6)	5·1 (3·6–7·3)	6·8 (4·9–9·7)	7·5 (5·4–10·7)	5·7 (4·1–8·2)
Saint Vincent and the Grenadines	5·8 (4·1–8·3)	6·4 (4·6–9·1)	4·9 (3·4–7·1)	6·3 (4·6–9·1)	7·0 (5·0–10·1)	5·3 (3·8–7·6)
Suriname	5·7 (3·9–8·2)	6·2 (4·2–8·9)	4·8 (3·3–7·0)	6·0 (4·3–8·6)	6·6 (4·7–9·3)	5·1 (3·5–7·3)
Trinidad and Tobago	5·9 (4·1–8·5)	6·4 (4·5–9·3)	5·2 (3·6–7·4)	6·7 (4·8–9·6)	7·4 (5·2–10·6)	5·8 (4·0–8·4)
United States Virgin Islands	6·8 (4·8–9·8)	7·4 (5·2–10·6)	5·7 (4·0–8·3)	7·5 (5·4–10·6)	8·2 (6·0–11·4)	6·3 (4·5–9·0)
**Central Latin America**	**7·1 (5·5–9·3)**	**7·8 (6·1–10·3)**	**5·8 (4·5–7·6)**	**7·7 (6·1–9·8)**	**8·4 (6·6–10·8)**	**6·2 (4·9–8·1)**
Colombia	7·3 (5·3–10·3)	8·1 (5·7–11·4)	6·1 (4·4–8·7)	8·4 (6·0–11·8)	9·6 (6·9–13·3)	7·1 (5·1–10·0)
Costa Rica	8·2 (5·7–11·6)	9·1 (6·4–13·0)	6·7 (4·7–9·6)	8·9 (6·4–12·7)	9·9 (7·1–14·2)	7·2 (5·2–10·2)
El Salvador	7·2 (5·0–10·1)	7·7 (5·4–11·0)	5·8 (4·2–8·4)	7·8 (5·6–11·1)	8·5 (6·1–12·1)	6·3 (4·5–8·7)
Guatemala	6·9 (4·9–10·0)	7·6 (5·3–10·9)	5·6 (4·0–8·0)	7·2 (5·1–10·3)	7·9 (5·6–11·2)	5·8 (4·1–8·2)
Honduras	6·6 (4·7–9·4)	7·2 (5·1–10·4)	5·3 (3·8–7·5)	6·9 (4·9–9·9)	7·6 (5·4–10·9)	5·5 (3·9–7·7)
Mexico	6·9 (5·8–8·4)	7·6 (6·4–9·3)	5·6 (4·7–6·8)	7·5 (6·3–9·2)	8·2 (6·8–10·0)	6·0 (5·0–7·3)
Nicaragua	6·7 (4·6–9·6)	7·3 (4·9–10·5)	5·5 (3·8–7·9)	7·7 (5·5–11·2)	8·5 (6·0–12·3)	6·3 (4·4–9·1)
Panama	8·0 (5·7–11·3)	8·9 (6·4–12·7)	6·6 (4·7–9·2)	8·4 (6·1–11·8)	9·4 (6·8–13·3)	6·8 (4·9–9·5)

Venezuela (Bolivarian Republic of)	7·5 (5·3–10·7)	8·4 (5·9–12·0)	6·0 (4·2–8·6)	8·1 (5·8–11·4)	9·0 (6·6–12·9)	6·6 (4·7–9·2)
**Tropical Latin America**	**13·6 (11·1–16·8)**	**14·8 (12·1–18·3)**	**11·0 (9·0–13·4)**	**16·0 (13·1–19·9)**	**17·3 (14·1–21·6)**	**12·7 (10·5–15·8)**
Brazil	13·6 (11·1–16·7)	14·8 (12·1–18·2)	11·0 (9·0–13·4)	16·0 (13·1–19·9)	17·4 (14·2–21·6)	12·8 (10·5–15·8)
Paraguay	13·7 (9·6–19·7)	14·6 (10·3–20·9)	11·0 (7·6–15·8)	14·7 (10·2–21·4)	15·7 (10·8–23·0)	11·8 (8·4–17·0)
**North Africa and Middle East**	**4·3 (3·0–6·1)**	**4·8 (3·3–6·8)**	**3·6 (2·5–5·1)**	**5·2 (3·6–7·3)**	**5·7 (4·1–8·1)**	**4·3 (3·0–6·1)**
Afghanistan	3·5 (2·3–5·3)	4·0 (2·6–6·0)	2·9 (1·9–4·4)	3·7 (2·4–5·6)	4·1 (2·6–6·2)	3·1 (2·0–4·7)
Algeria	4·4 (2·9–6·5)	4·9 (3·3–7·2)	3·6 (2·4–5·4)	5·5 (3·7–7·9)	6·1 (4·1–8·8)	4·4 (3·0–6·4)
Bahrain	4·8 (3·0–7·3)	5·4 (3·3–8·2)	4·3 (2·6–6·6)	5·5 (3·6–8·3)	6·2 (4·1–9·3)	5·0 (3·2–7·6)
Egypt	4·1 (2·8–6·2)	4·5 (3·0–6·8)	3·3 (2·3–4·9)	4·8 (3·2–7·0)	5·3 (3·5–7·7)	3·9 (2·6–5·7)
Iran (Islamic Republic of)	4·6 (3·7–5·7)	5·1 (4·1–6·3)	3·8 (3·1–4·7)	6·0 (4·8–7·4)	6·6 (5·3–8·2)	4·9 (4·0–6·2)
Iraq	4·0 (2·6–5·9)	4·3 (2·9–6·4)	3·4 (2·2–5·0)	5·1 (3·3–7·6)	5·7 (3·7–8·5)	4·3 (2·8–6·3)
Jordan	4·5 (2·9–6·7)	4·9 (3·2–7·4)	3·6 (2·3–5·5)	5·3 (3·5–7·8)	5·9 (3·9–8·6)	4·4 (2·9–6·6)
Kuwait	5·6 (3·5–8·5)	6·3 (3·9–9·5)	5·0 (3·1–7·5)	6·8 (4·3–10·2)	7·6 (4·8–11·5)	5·7 (3·7–8·6)
Lebanon	5·6 (4·0–7·9)	6·3 (4·4–9·0)	4·4 (3·1–6·1)	6·5 (4·3–9·5)	7·3 (4·9–10·6)	5·1 (3·4–7·4)
Libya	4·5 (2·9–6·7)	4·9 (3·2–7·5)	3·7 (2·4–5·6)	5·3 (3·5–7·8)	5·9 (3·9–8·7)	4·4 (2·9–6·5)
Morocco	4·3 (2·9–6·4)	4·8 (3·2–7·1)	3·6 (2·4–5·3)	4·9 (3·4–7·1)	5·5 (3·8–8·0)	4·1 (2·8–6·0)
Oman	4·3 (2·7–6·4)	4·7 (3·0–6·8)	4·0 (2·5–5·9)	5·5 (3·5–8·3)	6·1 (4·0–9·3)	5·0 (3·1–7·6)
Occupied Palestinian territory, including east Jerusalem	4·1 (2·7–6·2)	4·7 (3·1–7·1)	3·5 (2·3–5·3)	4·7 (3·2–6·9)	5·4 (3·6–7·9)	4·0 (2·7–6·0)
Qatar	5·2 (3·2–8·0)	5·9 (3·6–8·9)	4·8 (2·9–7·6)	6·3 (3·9–9·7)	7·4 (4·5–11·2)	5·8 (3·5–8·9)
Saudi Arabia	3·8 (2·4–5·7)	4·2 (2·7–6·3)	3·3 (2·1–5·0)	4·8 (3·2–7·2)	5·4 (3·6–8·1)	4·2 (2·7–6·3)
Sudan	3·8 (2·4–5·6)	4·1 (2·7–6·3)	3·2 (2·1–4·8)	4·3 (2·7–6·4)	4·8 (3·1–7·3)	3·6 (2·3–5·4)
Syrian Arab Republic	4·2 (2·7–6·3)	4·7 (3·0–6·9)	3·5 (2·3–5·4)	5·0 (3·4–7·2)	5·6 (3·8–8·0)	4·0 (2·8–5·9)
Tunisia	4·8 (3·4–7·0)	5·4 (3·8–7·9)	4·0 (2·7–5·7)	5·6 (3·9–8·1)	6·3 (4·4–9·2)	4·6 (3·2–6·5)
Türkiye	4·6 (3·1–6·9)	5·0 (3·4–7·5)	3·7 (2·5–5·5)	5·5 (3·8–7·9)	6·1 (4·1–8·8)	4·5 (3·1–6·4)
United Arab Emirates	4·6 (2·8–7·0)	5·2 (3·1–8·0)	4·2 (2·6–6·5)	5·7 (3·3–8·7)	6·5 (3·9–10·0)	5·3 (3·1–8·1)
Yemen	3·8 (2·4–5·6)	4·2 (2·7–6·2)	3·1 (2·0–4·7)	4·3 (2·8–6·5)	4·8 (3·1–7·3)	3·6 (2·3–5·4)
**South Asia**	**5·1 (4·1–6·4)**	**5·7 (4·6–7·1)**	**4·3 (3·5–5·4)**	**5·7 (4·6–7·2)**	**6·4 (5·2–8·0)**	**4·8 (3·9–6·0)**
Bangladesh	4·9 (3·2–7·3)	5·3 (3·5–8·0)	4·2 (2·8–6·1)	6·0 (4·2–8·6)	6·6 (4·5–9·4)	5·0 (3·6–7·1)
Bhutan	4·9 (3·4–7·1)	5·5 (3·8–8·0)	4·2 (2·9–6·1)	5·9 (4·2–8·5)	6·6 (4·7–9·5)	5·0 (3·5–7·3)

India	5·2 (4·2–6·4)	5·7 (4·7–7·1)	4·4 (3·6–5·4)	5·7 (4·7–7·1)	6·4 (5·3–7·9)	4·8 (4·0–6·0)
Nepal	5·0 (3·5–7·2)	5·5 (3·8–7·9)	4·2 (3·0–6·1)	5·6 (3·9–8·2)	6·2 (4·3–9·0)	4·6 (3·2–6·6)
Pakistan	4·9 (4·0–6·0)	5·4 (4·5–6·7)	4·1 (3·4–5·1)	5·4 (4·5–6·8)	6·1 (5·0–7·6)	4·5 (3·7–5·6)
**Southeast Asia, East Asia, and Oceania**	**2·1 (1·7–2·6)**	**2·3 (1·9–2·9)**	**1·7 (1·4–2·1)**	**2·3 (1·9–2·9)**	**2·6 (2·1–3·2)**	**1·9 (1·5–2·4)**
**East Asia**	**2·1 (1·8–2·6)**	**2·4 (2·0–2·9)**	**1·8 (1·4–2·2)**	**2·5 (2·0–3·0)**	**2·7 (2·2–3·4)**	**2·0 (1·6–2·4)**
China	2·1 (1·8–2·6)	2·4 (2·0–2·9)	1·8 (1·4–2·2)	2·5 (2·0–3·0)	2·7 (2·2–3·3)	2·0 (1·6–2·4)
Democratic People's Republic of Korea	2·1 (1·5–3·0)	2·2 (1·6–3·2)	1·6 (1·1–2·4)	2·3 (1·6–3·2)	2·5 (1·8–3·4)	1·8 (1·3–2·6)
Taiwan, China	2·5 (1·7–3·5)	2·7 (1·9–3·8)	2·0 (1·4–3·0)	2·8 (2·0–3·9)	3·0 (2·1–4·2)	2·2 (1·6–3·2)
**Oceania**	**1·6 (1·0–2·5)**	**1·8 (1·2–2·7)**	**1·4 (0·9–2·2)**	**1·7 (1·1–2·6)**	**2·0 (1·3–2·9)**	**1·5 (0·9–2·3)**
American Samoa	1·8 (1·1–2·6)	2·0 (1·3–2·9)	1·5 (0·9–2·2)	1·9 (1·3–2·7)	2·1 (1·5–3·0)	1·6 (1·1–2·3)
Cook Islands	2·1 (1·4–3·0)	2·3 (1·6–3·4)	1·8 (1·2–2·6)	2·3 (1·6–3·3)	2·5 (1·8–3·7)	1·9 (1·4–2·8)
Fiji	1·7 (1·1–2·5)	1·9 (1·2–2·7)	1·4 (0·9–2·2)	1·8 (1·2–2·6)	2·0 (1·4–2·9)	1·6 (1·0–2·3)
Guam	2·1 (1·4–3·1)	2·4 (1·6–3·4)	1·8 (1·2–2·7)	2·1 (1·5–3·0)	2·4 (1·7–3·3)	1·8 (1·2–2·6)
Kiribati	1·5 (1·0–2·3)	1·7 (1·1–2·6)	1·3 (0·8–2·1)	1·6 (1·1–2·4)	1·8 (1·2–2·6)	1·4 (0·9–2·2)
Marshall Islands	1·5 (0·9–2·2)	1·6 (1·0–2·4)	1·3 (0·8–2·0)	1·7 (1·1–2·5)	1·9 (1·3–2·8)	1·5 (0·9–2·2)
Micronesia (Federated States of)	1·5 (1·0–2·3)	1·7 (1·1–2·6)	1·3 (0·8–2·0)	1·7 (1·1–2·5)	1·9 (1·3–2·8)	1·5 (1·0–2·2)
Nauru	1·6 (1·0–2·4)	1·8 (1·2–2·7)	1·4 (0·9–2·1)	1·7 (1·1–2·5)	1·9 (1·3–2·8)	1·5 (0·9–2·3)
Niue	1·9 (1·3–2·7)	2·1 (1·5–3·0)	1·6 (1·1–2·4)	1·9 (1·4–2·8)	2·2 (1·5–3·1)	1·6 (1·1–2·5)
Northern Mariana Islands	2·1 (1·3–3·2)	2·3 (1·4–3·7)	1·9 (1·1–2·9)	2·1 (1·4–2·9)	2·4 (1·6–3·4)	1·8 (1·2–2·6)
Palau	1·9 (1·3–2·9)	2·2 (1·5–3·2)	1·7 (1·1–2·6)	2·0 (1·4–3·0)	2·3 (1·6–3·3)	1·8 (1·2–2·7)
Papua New Guinea	1·6 (1·0–2·4)	1·8 (1·1–2·7)	1·4 (0·8–2·1)	1·7 (1·1–2·6)	1·9 (1·2–2·9)	1·5 (0·9–2·3)
Samoa	1·7 (1·1–2·5)	1·9 (1·3–2·8)	1·4 (0·9–2·2)	1·8 (1·2–2·6)	2·0 (1·3–2·9)	1·5 (1·0–2·2)
Solomon Islands	1·5 (1·0–2·3)	1·7 (1·1–2·6)	1·3 (0·8–2·1)	1·7 (1·1–2·5)	1·9 (1·2–2·8)	1·4 (0·9–2·2)
Tokelau	1·8 (1·2–2·6)	2·0 (1·4–2·9)	1·5 (1·0–2·2)	1·9 (1·3–2·7)	2·1 (1·5–3·0)	1·6 (1·1–2·3)
Tonga	1·7 (1·1–2·4)	1·9 (1·3–2·7)	1·4 (0·9–2·1)	1·8 (1·2–2·7)	2·0 (1·4–3·0)	1·5 (1·0–2·2)
Tuvalu	1·7 (1·2–2·5)	2·0 (1·4–2·8)	1·5 (1·0–2·2)	1·8 (1·2–2·6)	2·0 (1·4–2·9)	1·5 (1·0–2·3)
Vanuatu	1·5 (1·0–2·3)	1·7 (1·1–2·6)	1·3 (0·8–2·1)	1·6 (1·1–2·4)	1·8 (1·2–2·7)	1·4 (0·9–2·1)
**Southeast Asia**	**1·8 (1·3–2·4)**	**2·0 (1·5–2·6)**	**1·5 (1·1–2·0)**	**2·0 (1·6–2·7)**	**2·2 (1·7–3·0)**	**1·7 (1·3–2·3)**
Cambodia	1·5 (0·9–2·2)	1·6 (1·1–2·5)	1·2 (0·8–1·9)	1·8 (1·2–2·6)	2·0 (1·3–2·9)	1·5 (1·0–2·3)

Indonesia	1·6 (1·3–2·0)	1·8 (1·4–2·3)	1·4 (1·1–1·7)	1·9 (1·5–2·4)	2·1 (1·7–2·6)	1·6 (1·3–2·0)
Lao People's Democratic Republic	1·4 (0·9–2·1)	1·5 (1·0–2·3)	1·2 (0·8–1·8)	1·6 (1·0–2·5)	1·8 (1·2–2·7)	1·5 (0·9–2·3)
Malaysia	1·8 (1·2–2·6)	2·0 (1·4–2·9)	1·6 (1·0–2·3)	2·1 (1·5–3·1)	2·3 (1·6–3·4)	1·9 (1·3–2·8)
Maldives	1·6 (1·1–2·5)	1·8 (1·1–2·7)	1·4 (0·9–2·1)	2·4 (1·6–3·5)	2·6 (1·7–3·8)	2·2 (1·4–3·3)
Mauritius	2·1 (1·4–3·1)	2·2 (1·5–3·3)	1·8 (1·2–2·7)	2·2 (1·6–3·2)	2·4 (1·7–3·5)	1·9 (1·3–2·8)
Myanmar	1·5 (1·0–2·2)	1·6 (1·1–2·5)	1·3 (0·8–2·0)	1·7 (1·2–2·5)	1·9 (1·3–2·8)	1·5 (1·0–2·2)
Philippines	1·7 (1·4–2·2)	1·9 (1·5–2·4)	1·5 (1·2–1·8)	1·8 (1·5–2·3)	2·0 (1·6–2·5)	1·6 (1·3–2·0)
Seychelles	1·8 (1·2–2·7)	2·0 (1·4–2·9)	1·6 (1·1–2·4)	2·1 (1·4–3·0)	2·3 (1·6–3·3)	1·9 (1·3–2·7)
Sri Lanka	1·8 (1·2–2·7)	2·0 (1·3–3·0)	1·6 (1·1–2·4)	2·2 (1·5–3·1)	2·4 (1·6–3·4)	2·0 (1·3–2·8)
Thailand	2·0 (1·3–2·9)	2·3 (1·5–3·2)	1·7 (1·1–2·5)	2·4 (1·7–3·5)	2·7 (1·9–3·8)	2·1 (1·5–3·0)
Timor-Leste	1·5 (1·0–2·2)	1·7 (1·1–2·5)	1·3 (0·9–2·0)	1·6 (1·0–2·3)	1·7 (1·1–2·6)	1·4 (0·9–2·1)
Viet Nam	1·9 (1·3–2·8)	2·1 (1·4–3·0)	1·6 (1·0–2·3)	2·3 (1·6–3·4)	2·5 (1·7–3·6)	2·0 (1·3–2·9)
**Sub-Saharan Africa**	**1·8 (1·4–2·4)**	**2·0 (1·5–2·7)**	**1·5 (1·2–2·1)**	**2·0 (1·5–2·6)**	**2·2 (1·6–2·9)**	**1·7 (1·2–2·2)**
**Central Sub-Saharan Africa**	**1·8 (1·2–2·7)**	**2·1 (1·3–3·1)**	**1·5 (0·9–2·2)**	**1·9 (1·2–2·9)**	**2·2 (1·4–3·3)**	**1·6 (1·0–2·4)**
Angola	1·8 (1·1–2·8)	2·1 (1·3–3·2)	1·5 (0·9–2·2)	2·0 (1·2–2·9)	2·2 (1·4–3·3)	1·6 (1·0–2·3)
Central African Republic	1·7 (1·1–2·7)	2·0 (1·3–3·0)	1·4 (0·9–2·2)	1·8 (1·1–2·8)	2·1 (1·3–3·1)	1·5 (0·9–2·3)
Congo	1·8 (1·2–2·8)	2·1 (1·4–3·2)	1·5 (0·9–2·2)	2·1 (1·4–3·1)	2·4 (1·5–3·5)	1·7 (1·1–2·5)
Democratic Republic of the Congo	1·8 (1·2–2·7)	2·1 (1·3–3·1)	1·5 (0·9–2·2)	1·9 (1·2–2·9)	2·2 (1·4–3·3)	1·6 (1·0–2·3)
Equatorial Guinea	1·8 (1·2–2·8)	2·1 (1·3–3·2)	1·4 (0·9–2·1)	2·1 (1·3–3·1)	2·4 (1·5–3·7)	1·6 (1·0–2·5)
Gabon	2·0 (1·3–2·8)	2·2 (1·5–3·2)	1·6 (1·1–2·4)	2·1 (1·4–3·1)	2·4 (1·6–3·5)	1·7 (1·2–2·5)
**Eastern Sub-Saharan Africa**	**1·8 (1·4–2·4)**	**2·1 (1·6–2·7)**	**1·5 (1·1–1·9)**	**2·0 (1·5–2·6)**	**2·2 (1·7–3·0)**	**1·6 (1·2–2·1)**
Burundi	1·8 (1·2–2·6)	2·1 (1·4–3·0)	1·5 (1·0–2·1)	1·9 (1·3–2·7)	2·1 (1·4–3·1)	1·6 (1·0–2·3)
Comoros	1·9 (1·3–2·8)	2·2 (1·5–3·2)	1·5 (1·0–2·3)	2·1 (1·5–3·0)	2·4 (1·7–3·4)	1·7 (1·2–2·5)
Djibouti	1·9 (1·2–2·7)	2·1 (1·4–3·1)	1·5 (1·0–2·2)	2·1 (1·4–3·1)	2·5 (1·6–3·6)	1·7 (1·1–2·5)
Eritrea	1·8 (1·2–2·7)	2·1 (1·4–3·1)	1·4 (0·9–2·0)	2·0 (1·3–2·9)	2·2 (1·5–3·2)	1·6 (1·0–2·3)
Ethiopia	1·8 (1·4–2·2)	2·0 (1·7–2·5)	1·4 (1·2–1·8)	1·9 (1·6–2·4)	2·3 (1·8–2·8)	1·6 (1·3–2·0)
Kenya	2·0 (1·6–2·4)	2·2 (1·8–2·7)	1·6 (1·3–2·0)	2·1 (1·7–2·5)	2·4 (1·9–2·9)	1·7 (1·3–2·1)
Madagascar	1·8 (1·3–2·7)	2·1 (1·4–3·1)	1·5 (1·0–2·2)	1·9 (1·3–2·8)	2·2 (1·5–3·2)	1·5 (1·0–2·3)
Malawi	1·8 (1·2–2·7)	2·1 (1·4–3·0)	1·5 (0·9–2·2)	1·9 (1·3–2·7)	2·1 (1·4–3·1)	1·5 (1·0–2·2)

Mozambique	1·9 (1·3–2·8)	2·1 (1·4–3·1)	1·5 (1·0–2·2)	1·8 (1·2–2·7)	2·1 (1·4–3·0)	1·5 (1·0–2·2)
Rwanda	1·8 (1·2–2·7)	2·0 (1·3–2·9)	1·4 (0·9–2·1)	2·1 (1·4–3·0)	2·3 (1·6–3·3)	1·6 (1·1–2·4)
Somalia	1·8 (1·2–2·6)	2·0 (1·3–2·9)	1·3 (0·9–2·0)	1·7 (1·1–2·6)	2·0 (1·3–2·9)	1·4 (0·8–2·1)
South Sudan	1·8 (1·2–2·7)	2·1 (1·4–3·0)	1·5 (1·0–2·2)	1·9 (1·3–2·7)	2·1 (1·4–3·1)	1·5 (1·0–2·2)
Uganda	1·7 (1·1–2·5)	1·9 (1·3–2·8)	1·4 (0·9–2·0)	2·0 (1·3–2·8)	2·2 (1·5–3·2)	1·6 (1·0–2·3)
United Republic of Tanzania	1·9 (1·3–2·7)	2·2 (1·5–3·1)	1·5 (1·0–2·2)	2·0 (1·4–2·9)	2·3 (1·6–3·3)	1·6 (1·1–2·3)
Zambia	1·7 (1·2–2·5)	2·0 (1·3–2·9)	1·4 (0·9–2·1)	1·9 (1·3–2·9)	2·2 (1·4–3·2)	1·6 (1·0–2·4)
**Southern Sub-Saharan Africa**	**2·5 (2·0–3·1)**	**3·1 (2·5–4·0)**	**1·2 (0·9–1·6)**	**2·6 (2·1–3·3)**	**3·3 (2·7–4·3)**	**1·4 (1·1–1·8)**
Botswana	2·3 (1·5–3·4)	3·0 (2·0–4·2)	1·2 (0·8–1·8)	2·6 (1·7–3·9)	3·4 (2·3–5·0)	1·4 (0·9–2·2)
Eswatini	2·1 (1·4–3·2)	2·8 (1·9–4·1)	1·1 (0·7–1·7)	2·2 (1·5–3·3)	2·9 (2·0–4·2)	1·2 (0·7–1·8)
Lesotho	2·3 (1·6–3·4)	2·9 (2·0–4·1)	1·2 (0·7–1·7)	2·2 (1·5–3·2)	2·8 (2·0–4·1)	1·2 (0·7–1·8)
Namibia	2·2 (1·5–3·1)	2·9 (2·1–4·1)	1·2 (0·8–1·8)	2·4 (1·6–3·5)	3·1 (2·1–4·5)	1·3 (0·8–2·0)
South Africa	2·5 (2·1–3·1)	3·2 (2·6–3·9)	1·2 (1·0–1·5)	2·7 (2·2–3·3)	3·4 (2·8–4·2)	1·4 (1·1–1·8)
Zimbabwe	2·2 (1·6–3·2)	2·9 (2·0–4·1)	1·2 (0·8–1·8)	2·2 (1·5–3·3)	2·9 (2·0–4·3)	1·2 (0·8–1·8)
**Western Sub-Saharan Africa**	**1·7 (1·3–2·3)**	**1·7 (1·3–2·2)**	**1·8 (1·4–2·4)**	**1·8 (1·4–2·5)**	**1·8 (1·3–2·4)**	**1·9 (1·5–2·6)**
Benin	1·7 (1·2–2·5)	1·7 (1·1–2·4)	1·8 (1·2–2·6)	1·8 (1·2–2·6)	1·7 (1·2–2·5)	1·9 (1·3–2·8)
Burkina Faso	1·7 (1·2–2·5)	1·7 (1·1–2·4)	1·8 (1·3–2·6)	1·8 (1·2–2·6)	1·7 (1·2–2·5)	1·9 (1·3–2·8)
Cabo Verde	2·0 (1·4–2·8)	1·9 (1·4–2·7)	2·1 (1·4–3·0)	2·3 (1·6–3·2)	2·2 (1·5–3·0)	2·4 (1·6–3·5)
Cameroon	1·7 (1·2–2·5)	1·7 (1·1–2·4)	1·8 (1·2–2·7)	1·8 (1·3–2·7)	1·8 (1·2–2·7)	2·0 (1·3–2·8)
Chad	1·7 (1·2–2·4)	1·6 (1·1–2·4)	1·7 (1·2–2·6)	1·7 (1·1–2·4)	1·6 (1·1–2·4)	1·8 (1·2–2·5)
Côte d'Ivoire	1·7 (1·1–2·4)	1·6 (1·1–2·3)	1·8 (1·2–2·6)	1·9 (1·3–2·7)	1·8 (1·2–2·6)	2·0 (1·4–2·9)
Gambia	1·7 (1·2–2·6)	1·7 (1·1–2·6)	1·8 (1·3–2·6)	1·8 (1·2–2·6)	1·7 (1·2–2·5)	1·9 (1·3–2·7)
Ghana	1·8 (1·3–2·7)	1·8 (1·2–2·6)	1·9 (1·3–2·8)	1·9 (1·3–2·8)	1·8 (1·3–2·8)	2·0 (1·4–2·9)
Guinea	1·7 (1·2–2·4)	1·7 (1·2–2·4)	1·8 (1·3–2·5)	1·7 (1·2–2·5)	1·7 (1·1–2·5)	1·8 (1·3–2·6)
Guinea-Bissau	1·6 (1·1–2·4)	1·5 (1·0–2·3)	1·7 (1·1–2·4)	1·7 (1·1–2·5)	1·7 (1·1–2·5)	1·8 (1·2–2·6)
Liberia	1·7 (1·2–2·4)	1·6 (1·1–2·3)	1·8 (1·3–2·6)	1·8 (1·2–2·7)	1·8 (1·2–2·7)	2·0 (1·3–2·9)
Mali	1·7 (1·2–2·5)	1·6 (1·1–2·4)	1·8 (1·3–2·5)	1·7 (1·2–2·5)	1·6 (1·1–2·4)	1·8 (1·2–2·6)
Mauritania	1·8 (1·3–2·6)	1·8 (1·2–2·5)	1·9 (1·3–2·7)	1·9 (1·4–2·8)	1·9 (1·3–2·7)	2·0 (1·4–2·9)
Niger	1·7 (1·1–2·4)	1·6 (1·0–2·3)	1·8 (1·2–2·6)	1·7 (1·2–2·5)	1·6 (1·1–2·4)	1·8 (1·2–2·6)

Nigeria	1·7 (1·4–2·1)	1·6 (1·3–2·0)	1·8 (1·5–2·2)	1·8 (1·5–2·3)	1·8 (1·4–2·2)	1·9 (1·6–2·4)
Sao Tome and Principe	1·7 (1·2–2·4)	1·7 (1·2–2·4)	1·7 (1·2–2·5)	1·9 (1·3–2·8)	1·9 (1·3–2·7)	2·0 (1·4–3·0)
Senegal	1·7 (1·2–2·5)	1·7 (1·1–2·5)	1·8 (1·3–2·6)	1·8 (1·3–2·7)	1·8 (1·2–2·6)	1·9 (1·3–2·8)
Sierra Leone	1·8 (1·2–2·5)	1·7 (1·2–2·4)	1·9 (1·3–2·6)	1·8 (1·2–2·7)	1·8 (1·2–2·6)	1·9 (1·4–2·8)
Togo	1·7 (1·2–2·5)	1·7 (1·1–2·5)	1·8 (1·2–2·7)	1·9 (1·3–2·7)	1·8 (1·2–2·7)	1·9 (1·3–2·8)

Note: UI = Uncertainty interval

Table S6: Counts and proportions of major depressive disorder cases receiving minimally adequate treatment in 2000 and 2021 by location (95% UIs)

Location	2000			2021			Change in MAT utilisation (%)
	MDD cases (thousands)	Receiving MAT (thousands)	Receiving MAT (%)	MDD cases (thousands)	Receiving MAT (thousands)	Receiving MAT (%)	
**Global**	**149202·0 (133113·9–172795·8)**	**13689·7 (10721·7–17600·7)**	**9·2 (7·4–11·7)**	**234374·1 (204875·6–275724·6)**	**21253·2 (16319·2–27846·4)**	**9·1 (7·2–11·6)**	**-1·2 (-4·6–2·0)**
**Central Europe, Eastern Europe, and Central Asia**	**11036·9 (9762·5–12647·9)**	**1245·5 (960·2–1647·9)**	**11·3 (9·1–14·3)**	**13127·2 (11329·5–15203·9)**	**1655·0 (1254·0–2161·2)**	**12·6 (10·1–16·1)**	**11·7 (9·8–13·5)**
**Central Asia**	**1492·0 (1294·2–1739·0)**	**143·1 (101·0–210·8)**	**9·6 (7·0–13·3)**	**2461·0 (2033·6–2990·1)**	**266·9 (179·2–409·0)**	**10·8 (7·8–15·2)**	**13·0 (8·1–18·0)**
Armenia	62·4 (53·0–74·5)	6·7 (4·8–9·8)	10·8 (7·9–15·0)	91·4 (68·9–118·3)	11·0 (7·0–16·9)	12·0 (8·7–16·6)	11·5 (4·9–18·9)
Azerbaijan	124·3 (104·4–149·3)	12·0 (8·1–17·5)	9·6 (6·8–13·6)	252·7 (186·9–327·4)	27·7 (17·4–43·4)	11·0 (7·9–15·9)	14·1 (5·5–23·8)
Georgia	127·1 (109·8–146·8)	14·0 (9·9–19·9)	11·0 (8·0–15·3)	134·5 (100·3–172·7)	15·4 (9·7–22·7)	11·4 (8·3–16·0)	3·9 (-2·1–10·6)
Kazakhstan	373·6 (327·3–433·3)	36·2 (26·0–53·5)	9·7 (7·0–13·5)	542·9 (404·1–699·6)	62·2 (39·5–94·9)	11·4 (8·2–15·9)	17·9 (11·1–25·1)
Kyrgyzstan	111·1 (94·5–130·5)	10·4 (7·2–15·2)	9·4 (6·7–13·1)	188·2 (144·4–238·3)	20·0 (12·6–31·8)	10·6 (7·5–15·0)	13·6 (6·1–22·3)
Mongolia	65·0 (54·7–77·5)	5·6 (3·7–8·4)	8·7 (6·0–12·8)	99·8 (75·9–131·4)	10·6 (6·5–17·1)	10·6 (7·4–15·1)	21·8 (9·6–34·3)
Tajikistan	86·2 (72·0–104·2)	7·6 (5·2–11·2)	8·8 (6·2–12·6)	198·4 (148·5–263·7)	19·5 (11·9–33·0)	9·8 (7·0–14·1)	11·4 (3·5–19·8)
Turkmenistan	75·4 (64·2–90·4)	6·8 (4·6–10·0)	9·0 (6·4–12·8)	122·2 (89·8–158·5)	12·1 (7·7–19·0)	9·9 (7·0–14·1)	10·3 (1·8–19·0)
Uzbekistan	466·9 (398·8–554·9)	43·8 (29·6–64·5)	9·4 (6·6–13·3)	830·9 (621·4–1092·3)	88·5 (55·3–137·4)	10·6 (7·6–15·0)	13·8 (4·7–24·1)
**Central Europe**	**2454·8 (2155·6–2806·0)**	**305·5 (227·0–435·2)**	**12·4 (9·5–17·0)**	**2913·2 (2465·7–3397·5)**	**395·8 (287·6–547·3)**	**13·6 (10·4–18·2)**	**9·3 (5·6–13·3)**
Albania	42·2 (35·8–50·6)	4·7 (3·2–7·0)	11·0 (7·9–15·5)	58·3 (43·0–77·9)	7·4 (4·5–11·3)	12·6 (9·2–17·6)	14·8 (3·3–26·8)
Bosnia and Herzegovina	98·6 (84·8–114·7)	11·6 (8·1–16·8)	11·7 (8·6–16·6)	95·9 (71·5–127·2)	12·3 (7·5–19·3)	12·8 (9·4–17·9)	9·7 (0·9–18·1)
Bulgaria	196·3 (167·6–229·4)	17·0 (12·0–23·9)	8·7 (6·4–11·6)	213·8 (161·5–278·3)	20·3 (12·9–31·3)	9·5 (6·9–13·0)	9·3 (1·8–17·7)
Croatia	127·8 (111·2–149·6)	16·4 (11·5–24·6)	12·8 (9·4–18·1)	136·8 (101·1–178·2)	18·9 (12·2–28·8)	13·8 (10·1–19·1)	7·8 (0·4–14·5)
Czechia	264·8 (232·5–304·4)	34·7 (24·7–50·9)	13·1 (9·6–18·8)	317·4 (239·4–406·2)	44·7 (28·3–66·6)	14·1 (10·2–19·6)	7·6 (0·8–17·5)
Hungary	267·9 (233·6–303·8)	33·6 (24·3–49·4)	12·5 (9·2–17·7)	291·5 (215·9–375·5)	39·0 (25·5–60·8)	13·4 (9·8–18·7)	6·8 (-1·3–15·9)
Montenegro	13·0 (11·0–15·3)	1·6 (1·1–2·4)	12·4 (9·0–17·1)	17·8 (13·2–23·4)	2·4 (1·5–3·7)	13·5 (9·8–18·8)	8·7 (2·0–16·2)
North Macedonia	36·0 (30·7–43·2)	4·2 (3·0–6·2)	11·6 (8·5–16·5)	56·6 (43·3–73·4)	7·2 (4·7–11·0)	12·8 (9·3–17·8)	10·3 (4·0–17·0)
Poland	555·0 (473·9–650·0)	63·9 (50·0–79·9)	11·5 (9·6–14·1)	732·0 (609·2–854·4)	92·4 (70·6–115·0)	12·6 (10·6–15·2)	9·6 (6·6–12·9)
Romania	440·3 (374·0–523·8)	67·9 (48·7–102·4)	15·4 (11·4–21·5)	512·7 (389·1–661·0)	87·3 (55·9–135·3)	17·0 (12·4–24·0)	10·4 (3·2–18·5)
Serbia	229·2 (195·9–270·2)	26·9 (18·7–38·7)	11·7 (8·6–16·4)	256·5 (186·2–338·9)	32·8 (21·2–49·9)	12·8 (9·4–17·7)	9·6 (1·4–18·2)
Slovakia	118·0 (104·0–135·4)	14·3 (10·2–21·4)	12·1 (8·9–17·1)	153·1 (114·2–198·5)	20·6 (13·2–30·8)	13·4 (9·9–19·0)	10·8 (3·6–19·9)

Slovenia	65·7 (56·8–76·4)	8·7 (6·0–12·5)	13·2 (9·8–18·6)	70·8 (53·5–93·0)	10·6 (6·9–15·6)	15·0 (10·9–20·7)	13·3 (3·2–24·2)
**Eastern Europe**	**7090·1 (6136·9–8217·8)**	**796·9 (627·6–999·1)**	**11·2 (9·3–13·9)**	**7753·1 (6561·8–9030·7)**	**992·2 (767·4–1252·5)**	**12·8 (10·6–15·8)**	**13·9 (11·8–16·2)**
Belarus	380·5 (324·9–444·8)	44·8 (31·4–66·8)	11·8 (8·7–16·7)	461·8 (351·1–602·4)	62·4 (40·3–95·9)	13·5 (9·8–19·1)	14·8 (7·5–22·6)
Estonia	63·3 (56·7–71·3)	7·6 (5·4–11·0)	12·0 (8·7–16·8)	59·5 (46·0–79·0)	8·2 (5·2–12·0)	13·7 (10·0–19·0)	13·9 (6·9–22·2)
Latvia	94·4 (81·6–110·4)	11·2 (7·8–16·6)	11·8 (8·6–16·6)	90·2 (65·7–116·9)	11·7 (7·4–17·8)	12·9 (9·4–17·8)	9·6 (1·8–17·9)
Lithuania	143·2 (123·5–170·2)	17·4 (12·2–26·4)	12·2 (8·9–17·2)	146·2 (112·8–194·0)	18·9 (12·3–29·0)	12·9 (9·5–18·2)	6·3 (-0·6–14·7)
Republic of Moldova	116·5 (99·4–136·9)	13·0 (9·0–19·5)	11·1 (8·1–15·5)	130·4 (96·6–168·7)	16·0 (10·3–24·0)	12·3 (8·9–17·2)	10·5 (2·5–19·2)
Russian Federation	4086·8 (3483·6–4791·9)	443·7 (347·9–552·4)	10·9 (9·1–13·2)	4771·3 (4053·9–5605·1)	611·3 (471·4–762·8)	12·8 (10·8–15·6)	18·0 (15·9–20·2)
Ukraine	2205·4 (1870·0–2579·7)	259·1 (203·3–324·8)	11·8 (9·7–14·3)	2093·7 (1588·8–2677·1)	263·8 (191·6–357·0)	12·6 (10·6–15·3)	7·4 (1·6–12·8)
**High-income**	**30805·6 (28065·2–34521·3)**	**7911·5 (6273·4–10127·0)**	**25·7 (20·7–32·7)**	**41180·2 (36781·3–47862·6)**	**11139·5 (8618·4–14600·5)**	**27·0 (21·7–34·4)**	**5·3 (1·7–9·2)**
Australasia	848·7 (781·0–933·1)	215·7 (174·9–266·4)	25·4 (21·1–30·8)	1180·3 (927·4–1492·7)	345·0 (225·6–528·8)	29·2 (21·4–40·8)	14·4 (-6·0–42·3)
Australia	741·9 (684·0–817·1)	191·3 (155·2–239·1)	25·8 (21·4–31·2)	1034·2 (790·6–1355·0)	311·7 (197·2–493·4)	30·1 (21·6–42·6)	16·2 (-6·5–47·6)
New Zealand	106·7 (91·5–123·8)	24·3 (18·9–30·0)	22·8 (18·7–28·0)	146·1 (113·7–189·4)	33·3 (24·4–45·1)	22·8 (19·0–27·7)	0·2 (-5·1–5·9)
**High-income Asia Pacific**	**3127·1 (2812·7–3471·7)**	**773·0 (614·7–981·4)**	**24·7 (20·2–31·1)**	**3832·0 (3323·4–4411·4)**	**1012·2 (797·2–1292·4)**	**26·4 (21·9–32·4)**	**7·0 (1·0–13·5)**
Brunei Darussalam	3·0 (2·5–3·8)	0·6 (0·4–0·9)	19·3 (12·7–28·6)	5·6 (4·1–7·5)	1·2 (0·7–1·8)	21·2 (14·5–31·0)	10·0 (2·3–19·0)
Japan	2374·0 (2141·3–2649·9)	576·2 (468·6–718·0)	24·3 (20·5–29·2)	2726·8 (2367·3–3095·7)	682·8 (550·8–832·3)	25·0 (21·4–29·6)	3·2 (-1·3–8·3)
Republic of Korea	655·2 (585·5–731·5)	172·6 (120·1–243·0)	26·3 (18·5–37·5)	998·5 (759·6–1261·0)	299·7 (205·8–428·8)	30·0 (23·1–39·8)	14·9 (-2·6–31·6)
Singapore	94·9 (82·8–108·1)	23·5 (15·6–35·3)	24·8 (16·8–36·6)	101·2 (78·8–127·8)	28·6 (18·8–44·2)	28·2 (20·4–40·6)	14·4 (2·5–27·6)
**High-income North America**	**11667·8 (10438·0–13274·3)**	**3115·6 (2471·2–3919·7)**	**26·7 (22·0–32·6)**	**16201·9 (14444·5–18631·3)**	**4499·0 (3605·8–5634·8)**	**27·8 (22·8–34·3)**	**4·0 (0·4–8·0)**
Canada	806·5 (721·1–913·4)	268·6 (194·8–371·7)	33·3 (24·6–45·3)	1136·9 (855·9–1498·0)	411·5 (264·1–618·7)	36·2 (26·1–50·0)	8·8 (-6·8–32·2)
Greenland	3·3 (2·8–3·9)	0·9 (0·6–1·3)	25·8 (16·8–37·9)	3·8 (2·9–4·9)	1·0 (0·7–1·6)	26·9 (18·4–38·8)	4·6 (-4·1–13·8)
United States of America	10858·0 (9687·1–12399·5)	2846·2 (2276·3–3547·0)	26·2 (21·6–31·7)	15061·2 (13368·9–17190·7)	4086·5 (3310·2–5074·8)	27·1 (22·4–33·2)	3·6 (0·6–6·8)
**Southern Latin America**	**1354·5 (1207·0–1560·7)**	**289·4 (200·5–415·7)**	**21·4 (15·1–30·7)**	**2033·6 (1652·4–2496·3)**	**456·5 (300·9–681·0)**	**22·4 (16·0–31·6)**	**5·2 (-2·1–10·7)**
Argentina	747·9 (642·2–887·2)	154·6 (106·0–228·8)	20·7 (14·7–30·0)	1155·8 (886·5–1463·0)	241·0 (153·5–362·1)	20·8 (15·0–29·6)	1·2 (-11·1–11·3)
Chile	535·5 (486·5–596·8)	118·6 (81·5–170·1)	22·1 (15·3–31·7)	766·1 (574·5–1027·9)	188·8 (118·6–303·2)	24·6 (17·6–35·3)	11·4 (2·7–20·6)
Uruguay	71·1 (62·6–82·4)	16·2 (11·6–23·4)	22·8 (16·6–32·2)	111·8 (82·9–148·1)	26·7 (16·8–42·3)	23·9 (17·1–34·2)	4·6 (-2·4–11·6)
**Western Europe**	**13807·4 (12600·2–15278·7)**	**3517·9 (2730·0–4655·6)**	**25·5 (20·0–33·4)**	**17932·4 (15639·5–20954·7)**	**4826·8 (3574·5–6597·2)**	**26·9 (20·9–35·7)**	**5·5 (-0·1–11·2)**
Andorra	2·0 (1·7–2·5)	0·6 (0·4–0·8)	27·2 (19·1–39·3)	3·3 (2·4–4·4)	0·9 (0·6–1·4)	27·7 (19·7–40·0)	2·2 (-6·9–11·9)

Austria	196·8 (170·4–228·6)	50·8 (35·5–72·7)	25·8 (18·6–36·4)	246·2 (183·2–323·0)	65·9 (41·3–100·3)	26·7 (19·5–37·5)	3·9 (-2·8–11·4)
Belgium	280·9 (260·6–302·9)	91·1 (67·8–125·4)	32·4 (24·0–44·1)	410·0 (316·4–528·3)	141·1 (94·2–209·3)	34·4 (25·2–47·6)	6·0 (-2·4–17·8)
Cyprus	20·3 (16·9–24·8)	4·5 (3·1–6·7)	22·0 (15·6–31·9)	38·4 (28·6–51·7)	10·3 (6·3–15·9)	26·8 (19·0–38·7)	21·6 (15·1–29·0)
Denmark	174·6 (149·0–203·3)	43·3 (30·2–63·6)	24·8 (17·8–35·2)	200·7 (151·4–259·0)	52·2 (33·6–78·3)	26·0 (19·1–36·5)	5·3 (-2·0–12·3)
England	1868·8 (1602·2–2188·6)	456·9 (354·1–566·3)	24·5 (20·8–29·4)	2619·1 (2246·1–3065·4)	674·6 (528·4–832·1)	25·8 (21·7–31·2)	5·4 (3·3–7·3)
Finland	223·9 (201·2–249·5)	40·2 (31·9–51·4)	17·9 (14·8–22·2)	234·7 (180·9–301·0)	48·4 (31·1–74·7)	20·6 (15·2–29·0)	14·5 (-7·1–39·0)
France	2253·5 (2097·6–2449·1)	590·8 (425·5–830·4)	26·2 (19·0–37·0)	2655·0 (2051·9–3385·8)	771·2 (502·7–1218·8)	29·0 (21·3–40·7)	10·7 (1·2–24·0)
Germany	2179·0 (2011·5–2377·9)	717·3 (535·5–982·1)	32·9 (24·7–43·7)	2992·5 (2268·4–3907·3)	1048·6 (674·1–1574·4)	35·0 (26·0–48·0)	6·3 (-4·2–18·2)
Greece	698·1 (585·8–849·4)	178·9 (124·6–268·0)	25·6 (18·8–36·5)	605·3 (462·5–789·6)	154·8 (98·4–230·9)	25·6 (18·8–35·8)	0·1 (-7·7–7·3)
Iceland	6·3 (5·4–7·4)	1·6 (1·1–2·4)	25·9 (18·2–37·1)	8·5 (6·3–11·2)	2·3 (1·4–3·4)	26·6 (19·3–37·1)	3·0 (-4·7–11·0)
Ireland	134·3 (125·9–143·7)	35·9 (25·6–52·4)	26·7 (19·0–38·7)	217·4 (167·0–284·9)	65·6 (40·6–100·8)	30·1 (21·3–42·9)	12·8 (3·5–22·1)
Israel	242·6 (215·5–274·7)	43·6 (30·7–62·0)	18·0 (12·9–25·3)	369·7 (280·3–493·2)	73·8 (47·9–117·6)	20·0 (14·6–28·3)	11·2 (0·2–26·7)
Italy	2058·5 (1772·7–2390·5)	410·1 (323·1–499·7)	19·9 (17·0–23·9)	2406·0 (2038·3–2796·7)	468·8 (372·3–577·1)	19·5 (16·6–23·4)	-2·2 (-5·5–0·8)
Luxembourg	13·0 (11·3–15·2)	3·3 (2·3–5·1)	25·6 (18·3–37·0)	19·8 (15·1–25·6)	5·3 (3·6–8·1)	26·9 (19·3–38·2)	5·1 (-2·1–11·9)
Malta	9·2 (7·7–11·1)	2·2 (1·5–3·3)	23·6 (17·2–34·0)	12·4 (9·5–16·2)	3·2 (2·1–4·8)	26·3 (19·6–36·4)	11·5 (3·5–19·4)
Monaco	1·2 (1·0–1·5)	0·3 (0·2–0·5)	26·0 (19·3–36·8)	1·7 (1·2–2·2)	0·4 (0·3–0·7)	26·2 (19·4–36·7)	0·8 (-5·1–7·3)
Netherlands	471·5 (441·3–502·3)	147·0 (111·7–185·8)	31·2 (24·0–39·2)	627·9 (479·0–832·5)	190·4 (130·8–275·4)	30·3 (23·8–39·7)	-2·6 (-14·8–9·4)
Northern Ireland	65·4 (59·7–71·8)	16·0 (11·4–22·6)	24·5 (17·4–34·9)	89·0 (67·6–117·4)	23·2 (15·1–35·6)	26·1 (18·7–37·4)	6·6 (0·6–12·7)
Norway	127·7 (110·3–149·1)	32·5 (25·5–40·6)	25·5 (21·3–31·0)	191·3 (158·8–227·2)	51·1 (39·5–66·5)	26·7 (22·5–32·3)	5·1 (2·5–7·2)
Portugal	571·4 (472·5–686·3)	112·6 (78·1–173·3)	19·7 (14·4–27·6)	583·4 (443·3–758·0)	127·1 (82·5–188·3)	21·8 (16·1–30·5)	10·8 (2·6–19·4)
San Marino	1·0 (0·8–1·3)	0·3 (0·2–0·4)	27·5 (20·1–39·7)	1·5 (1·1–2·0)	0·4 (0·3–0·6)	27·3 (20·0–38·7)	-0·6 (-10·7–7·2)
Scotland	176·9 (159·2–198·1)	42·0 (30·4–61·1)	23·7 (17·3–34·1)	229·4 (173·0–302·2)	59·1 (36·5–91·4)	25·7 (18·6–36·8)	8·5 (1·8–15·1)
Spain	1312·8 (1194·1–1450·2)	305·8 (223·0–420·3)	23·3 (17·4–31·7)	2237·0 (1700·3–2870·6)	532·7 (347·6–792·4)	23·8 (17·9–33·3)	2·1 (-10·2–12·3)
Sweden	341·6 (305·7–385·5)	93·0 (67·2–136·1)	27·2 (19·9–38·2)	436·9 (351·5–541·1)	121·2 (81·6–183·5)	27·7 (20·5–39·3)	2·0 (-4·1–7·6)
Switzerland	267·5 (231·2–314·5)	71·2 (50·0–104·6)	26·6 (18·9–37·9)	359·5 (271·2–472·3)	100·2 (64·1–153·5)	27·9 (20·6–38·7)	5·0 (-2·6–13·2)
Wales	108·6 (95·1–124·3)	26·3 (19·0–37·9)	24·2 (17·7–34·4)	135·7 (102·9–176·6)	33·9 (22·0–51·6)	25·0 (18·3–35·0)	3·2 (-2·1–9·9)
**Latin America and Caribbean**	**11990·9 (10581·7–13937·3)**	**1212·2 (951·8–1578·1)**	**10·1 (8·2–12·8)**	**19772·4 (17219·3–23294·1)**	**2133·4 (1648·3–2786·0)**	**10·8 (8·8–13·6)**	**6·7 (2·3–11·8)**
**Andean Latin America**	**754·9 (654·9–886·3)**	**13·2 (8·9–19·5)**	**1·8 (1·2–2·5)**	**1615·0 (1299·8–2010·9)**	**33·0 (21·6–51·9)**	**2·0 (1·4–2·9)**	**16·6 (12·1–22·1)**
Bolivia (Plurinational State of)	175·9 (149·1–210·0)	2·9 (1·9–4·5)	1·7 (1·1–2·4)	394·2 (293·9–517·7)	7·5 (4·7–12·2)	1·9 (1·3–2·8)	14·3 (7·2–22·4)

Ecuador	258·9 (220·8–303·9)	4·6 (3·1–6·9)	1·8 (1·2–2·6)	559·7 (427·7–721·3)	11·3 (7·0–17·7)	2·0 (1·4–2·9)	13·5 (7·2–20·5)
Peru	320·1 (279·2–373·3)	5·7 (3·8–8·2)	1·8 (1·2–2·5)	661·0 (494·8–870·6)	14·1 (8·7–22·3)	2·1 (1·5–3·1)	20·9 (13·0–30·7)
**Caribbean**	**1047·9 (909·6–1234·2)**	**67·7 (47·2–100·6)**	**6·5 (4·6–9·2)**	**1577·9 (1290·4–1929·7)**	**104·9 (70·9–159·0)**	**6·6 (4·8–9·4)**	**3·0 (-2·4–9·1)**
Antigua and Barbuda	1·4 (1·2–1·8)	0·1 (0·1–0·1)	6·8 (4·8–9·8)	2·5 (1·8–3·3)	0·2 (0·1–0·3)	7·2 (5·1–10·4)	6·6 (-2·5–16·4)
Bahamas	5·8 (4·8–7·1)	0·4 (0·2–0·6)	6·5 (4·4–9·4)	11·1 (8·1–14·6)	0·8 (0·4–1·2)	6·8 (4·7–9·6)	4·6 (-4·6–13·6)
Barbados	5·7 (4·9–6·9)	0·4 (0·3–0·6)	7·1 (5·1–10·0)	9·9 (7·2–13·2)	0·7 (0·5–1·1)	7·3 (5·4–10·2)	4·2 (-4·2–12·0)
Belize	4·4 (3·6–5·3)	0·2 (0·2–0·4)	5·5 (3·8–8·1)	11·7 (8·8–15·5)	0·7 (0·4–1·1)	6·2 (4·3–9·0)	11·2 (4·5–19·9)
Bermuda	1·7 (1·5–2·1)	0·1 (0·1–0·2)	8·1 (5·9–11·3)	2·2 (1·6–2·8)	0·2 (0·1–0·3)	8·6 (6·2–12·2)	6·6 (-4·2–18·1)
Cuba	418·5 (360·6–495·2)	30·9 (20·9–46·3)	7·4 (5·3–10·5)	494·6 (377·1–641·2)	38·1 (24·6–58·6)	7·7 (5·6–10·9)	4·8 (-8·7–17·1)
Dominica	1·3 (1·1–1·6)	0·1 (0·1–0·1)	6·2 (4·5–8·7)	2·0 (1·5–2·6)	0·1 (0·1–0·2)	6·3 (4·5–9·0)	2·2 (-3·8–8·6)
Dominican Republic	223·2 (194·6–260·7)	13·5 (9·3–19·6)	6·0 (4·3–8·8)	367·0 (270·5–494·7)	23·2 (14·3–36·3)	6·3 (4·6–9·0)	4·7 (-1·7–10·8)
Grenada	2·2 (1·8–2·6)	0·1 (0·1–0·2)	5·9 (4·2–8·6)	3·1 (2·3–4·0)	0·2 (0·1–0·3)	6·4 (4·6–9·2)	8·0 (1·6–14·9)
Guyana	26·1 (22·0–30·8)	1·4 (0·9–2·1)	5·5 (3·7–8·1)	39·5 (29·7–51·9)	2·2 (1·4–3·6)	5·6 (3·9–8·2)	3·6 (-3·1–12·1)
Haiti	172·4 (141·6–209·3)	8·4 (5·5–13·1)	4·9 (3·3–7·0)	361·3 (268·1–482·1)	19·2 (11·7–30·9)	5·3 (3·6–7·8)	9·0 (2·8–15·3)
Jamaica	46·4 (38·5–57·1)	3·0 (2·0–4·5)	6·4 (4·5–9·2)	79·8 (58·7–107·8)	5·4 (3·4–8·6)	6·7 (4·8–9·7)	5·5 (-1·5–12·1)
Puerto Rico	77·1 (66·5–91·5)	5·4 (3·8–8·1)	7·0 (5·1–9·8)	88·4 (66·8–116·3)	7·0 (4·4–10·8)	7·9 (5·8–11·0)	12·8 (2·4–23·7)
Saint Kitts and Nevis	1·3 (1·0–1·6)	0·1 (0·1–0·1)	6·3 (4·4–9·0)	2·4 (1·7–3·3)	0·2 (0·1–0·3)	6·8 (4·8–9·9)	8·6 (-1·7–19·9)
Saint Lucia	3·2 (2·7–3·8)	0·2 (0·1–0·3)	6·1 (4·3–8·7)	6·2 (4·6–8·2)	0·4 (0·3–0·7)	6·8 (4·9–9·7)	12·4 (3·2–23·0)
Saint Vincent and the Grenadines	2·2 (1·9–2·7)	0·1 (0·1–0·2)	5·8 (4·1–8·3)	3·7 (2·8–4·8)	0·2 (0·1–0·4)	6·3 (4·6–9·1)	8·6 (-2·3–17·8)
Suriname	15·3 (12·9–17·8)	0·9 (0·6–1·3)	5·7 (3·9–8·2)	30·2 (22·9–39·5)	1·8 (1·1–2·8)	6·0 (4·3–8·6)	6·3 (-1·4–14·7)
Trinidad and Tobago	37·3 (31·5–44·4)	2·2 (1·5–3·2)	5·9 (4·1–8·5)	59·5 (44·4–79·5)	4·0 (2·5–6·6)	6·7 (4·8–9·6)	13·4 (5·3–23·0)
United States Virgin Islands	2·5 (2·1–3·1)	0·2 (0·1–0·3)	6·8 (4·8–9·8)	2·9 (2·2–3·8)	0·2 (0·1–0·3)	7·5 (5·4–10·6)	10·9 (-1·5–24·2)
**Central Latin America**	**3925·2 (3414·3–4568·3)**	**278·4 (210·2–374·9)**	**7·1 (5·5–9·3)**	**7868·0 (6823·6–9333·3)**	**605·0 (453·5–809·4)**	**7·7 (6·1–9·8)**	**8·5 (3·5–12·5)**
Colombia	661·9 (564·4–784·8)	48·2 (33·3–70·0)	7·3 (5·3–10·3)	893·5 (674·1–1168·6)	74·9 (47·3–115·1)	8·4 (6·0–11·8)	15·1 (3·8–27·2)
Costa Rica	83·7 (71·1–100·3)	6·8 (4·6–10·2)	8·2 (5·7–11·6)	157·5 (120·4–202·0)	14·0 (8·6–21·5)	8·9 (6·4–12·7)	9·3 (0·5–17·4)
El Salvador	140·8 (119·1–166·8)	10·1 (7·0–14·9)	7·2 (5·0–10·1)	203·0 (155·5–269·4)	15·9 (10·2–25·2)	7·8 (5·6–11·1)	9·2 (2·4–17·0)
Guatemala	263·9 (230·0–305·8)	18·3 (12·7–26·9)	6·9 (4·9–10·0)	509·3 (385·7–690·1)	37·0 (22·3–59·8)	7·2 (5·1–10·3)	4·6 (-1·8–11·4)
Honduras	101·0 (84·4–123·1)	6·7 (4·5–10·0)	6·6 (4·7–9·4)	274·5 (202·3–365·6)	19·1 (11·8–29·4)	6·9 (4·9–9·9)	5·3 (-1·3–12·9)
Mexico	2018·5 (1782·6–2342·3)	139·8 (112·1–174·2)	6·9 (5·8–8·4)	4752·5 (4098·9–5560·6)	357·0 (280·7–453·7)	7·5 (6·3–9·2)	8·4 (6·0–11·0)

Nicaragua	101·2 (83·3–121·6)	6·8 (4·5–10·1)	6·7 (4·6–9·6)	191·3 (138·8–246·8)	14·8 (9·3–23·4)	7·7 (5·5–11·2)	15·6 (8·0–25·0)
Panama	58·2 (48·8–69·8)	4·7 (3·2–6·9)	8·0 (5·7–11·3)	116·7 (86·5–154·5)	9·8 (6·2–15·3)	8·4 (6·1–11·8)	5·5 (-0·5–13·1)
Venezuela (Bolivarian Republic of)	496·0 (421·0–593·2)	37·2 (25·6–55·8)	7·5 (5·3–10·7)	769·7 (576·2–1011·5)	62·5 (39·2–96·9)	8·1 (5·8–11·4)	8·6 (-3·8–21·9)
**Tropical Latin America**	**6263·0 (5546·6–7237·0)**	**852·8 (677·9–1079·1)**	**13·6 (11·1–16·8)**	**8711·4 (7563·7–10125·5)**	**1390·6 (1091·7–1791·0)**	**16·0 (13·1–19·9)**	**17·3 (12·2–23·8)**
Brazil	6166·9 (5464·2–7127·8)	839·6 (667·3–1058·6)	13·6 (11·1–16·7)	8498·0 (7353·1–9873·6)	1359·3 (1073·4–1744·9)	16·0 (13·1–19·9)	17·5 (12·3–24·2)
Paraguay	96·1 (82·1–111·5)	13·2 (9·1–19·1)	13·7 (9·6–19·7)	213·4 (160·8–286·9)	31·3 (18·9–50·1)	14·7 (10·2–21·4)	6·8 (-0·0–13·8)
**North Africa and Middle East**	**12313·2 (10760·1–14572·2)**	**529·9 (363·3–762·3)**	**4·3 (3·0–6·1)**	**24394·1 (19957·8–29639·7)**	**1257·8 (833·1–1891·3)**	**5·2 (3·6–7·3)**	**19·8 (14·1–25·0)**
Afghanistan	458·3 (372·5–564·9)	16·2 (10·2–25·1)	3·5 (2·3–5·3)	1188·2 (866·5–1609·7)	43·6 (26·2–71·7)	3·7 (2·4–5·6)	4·0 (-5·4–14·4)
Algeria	893·7 (731·6–1118·3)	39·6 (25·2–60·8)	4·4 (2·9–6·5)	1541·8 (1145·7–2080·0)	84·1 (51·2–133·5)	5·5 (3·7–7·9)	23·2 (13·0–36·1)
Bahrain	27·4 (22·5–33·1)	1·3 (0·8–2·0)	4·8 (3·0–7·3)	73·8 (54·0–96·8)	4·1 (2·5–6·6)	5·5 (3·6–8·3)	15·9 (6·0–25·6)
Egypt	1622·0 (1489·2–1782·9)	66·1 (44·8–97·4)	4·1 (2·8–6·2)	3406·0 (2508·3–4459·7)	162·2 (96·8–263·5)	4·8 (3·2–7·0)	16·7 (7·5–26·7)
Iran (Islamic Republic of)	2191·7 (1839·2–2654·5)	101·2 (77·8–131·2)	4·6 (3·7–5·7)	4120·9 (3364·5–5009·0)	246·1 (179·7–322·5)	6·0 (4·8–7·4)	29·2 (22·8–36·1)
Iraq	519·2 (445·9–621·3)	20·5 (13·4–30·8)	4·0 (2·6–5·9)	1296·0 (972·5–1723·7)	66·4 (39·4–105·9)	5·1 (3·3–7·6)	29·7 (18·1–41·9)
Jordan	132·7 (108·2–165·9)	5·9 (3·7–9·4)	4·5 (2·9–6·7)	420·9 (310·0–566·9)	22·4 (13·9–35·3)	5·3 (3·5–7·8)	19·5 (8·3–30·1)
Kuwait	58·1 (47·7–72·2)	3·3 (2·0–5·3)	5·6 (3·5–8·5)	172·7 (124·3–234·9)	11·8 (6·8–19·5)	6·8 (4·3–10·2)	21·2 (6·1–35·9)
Lebanon	136·3 (119·2–155·4)	7·6 (5·3–10·9)	5·6 (4·0–7·9)	298·9 (217·8–404·4)	19·3 (11·8–31·4)	6·5 (4·3–9·5)	14·9 (4·6–24·4)
Libya	154·2 (124·9–192·5)	6·9 (4·3–10·9)	4·5 (2·9–6·7)	295·5 (222·4–395·7)	15·6 (9·4–25·1)	5·3 (3·5–7·8)	18·9 (4·2–34·0)
Morocco	1127·6 (950·9–1365·7)	48·9 (31·2–73·9)	4·3 (2·9–6·4)	1896·0 (1427·6–2437·1)	93·9 (58·2–151·0)	4·9 (3·4–7·1)	14·4 (6·0–23·4)
Oman	60·7 (49·0–76·4)	2·6 (1·6–4·2)	4·3 (2·7–6·4)	175·3 (128·2–237·6)	9·7 (5·6–16·3)	5·5 (3·5–8·3)	27·8 (17·0–40·1)
Occupied Palestinian territory, including east Jerusalem	139·7 (118·9–163·4)	5·8 (3·8–8·6)	4·1 (2·7–6·2)	291·9 (218·2–380·8)	13·8 (8·7–21·9)	4·7 (3·2–6·9)	14·8 (8·1–22·4)
Qatar	21·1 (16·9–26·4)	1·1 (0·7–1·8)	5·2 (3·2–8·0)	121·6 (89·4–166·9)	7·7 (4·3–12·9)	6·3 (3·9–9·7)	21·7 (15·2–29·4)
Saudi Arabia	557·7 (453·3–692·7)	21·1 (13·5–32·8)	3·8 (2·4–5·7)	1482·9 (1066·5–1990·9)	71·4 (42·2–113·4)	4·8 (3·2–7·2)	27·4 (14·6–40·7)
Sudan	747·1 (618·5–923·3)	28·1 (17·3–45·0)	3·8 (2·4–5·6)	1526·1 (1096·5–2090·8)	65·1 (37·8–104·0)	4·3 (2·7–6·4)	13·6 (7·1–20·3)
Syrian Arab Republic	416·4 (336·2–519·3)	17·5 (11·1–27·1)	4·2 (2·7–6·3)	540·3 (395·9–734·2)	27·1 (16·4–44·6)	5·0 (3·4–7·2)	19·5 (1·2–37·4)
Tunisia	393·9 (342·0–462·3)	18·9 (12·8–28·0)	4·8 (3·4–7·0)	715·5 (534·5–938·3)	40·2 (24·5–62·8)	5·6 (3·9–8·1)	16·6 (7·5–24·6)
Türkiye	2007·5 (1810·2–2243·4)	92·0 (61·2–137·1)	4·6 (3·1–6·9)	3294·2 (2465·5–4368·8)	181·4 (105·9–292·4)	5·5 (3·8–7·9)	20·4 (9·2–31·3)
United Arab Emirates	98·7 (79·9–119·2)	4·5 (2·7–7·4)	4·6 (2·8–7·0)	389·3 (281·4–533·5)	22·2 (11·6–36·6)	5·7 (3·3–8·7)	24·3 (0·5–47·5)
Yemen	549·5 (446·8–693·2)	20·6 (12·8–32·5)	3·8 (2·4–5·6)	1146·1 (839·5–1557·7)	49·6 (29·7–78·8)	4·3 (2·8–6·5)	15·3 (7·0–24·7)

**South Asia**	**36875·4 (32070·0–43429·9)**	**1884·3 (1438·1–2452·9)**	**5·1 (4·1–6·4)**	**60222·7 (51792·4–71109·4)**	**3445·9 (2654·5–4486·5)**	**5·7 (4·6–7·2)**	**12·0 (9·3–15·2)**
Bangladesh	3667·0 (3056·9–4353·9)	180·1 (116·0–268·6)	4·9 (3·2–7·3)	6874·4 (5234·0–8937·7)	412·2 (258·4–637·4)	6·0 (4·2–8·6)	22·7 (9·3–35·8)
Bhutan	15·2 (12·9–18·3)	0·8 (0·5–1·1)	4·9 (3·4–7·1)	23·0 (17·0–30·9)	1·4 (0·9–2·1)	5·9 (4·2–8·5)	19·3 (11·7–26·5)
India	29422·1 (25917·6–34651·3)	1518·8 (1183·4–1943·8)	5·2 (4·2–6·4)	45409·1 (39275·2–53483·2)	2599·6 (2065·1–3307·5)	5·7 (4·7–7·1)	11·0 (8·1–14·1)
Nepal	730·5 (641·8–853·6)	36·2 (24·5–52·7)	5·0 (3·5–7·2)	1417·8 (1042·8–1842·6)	79·1 (48·0–125·5)	5·6 (3·9–8·2)	12·7 (5·7–19·6)
Pakistan	3040·6 (2572·9–3638·7)	148·5 (111·9–190·8)	4·9 (4·0–6·0)	6498·3 (5139·7–8121·0)	353·6 (262·2–461·4)	5·4 (4·5–6·8)	11·4 (7·3–15·5)
**Southeast Asia, East Asia, and Oceania**	**27406·1 (24534·5–31583·6)**	**562·6 (444·8–719·0)**	**2·1 (1·7–2·6)**	**38947·7 (33825·2–45111·9)**	**900·8 (689·4–1158·4)**	**2·3 (1·9–2·9)**	**12·8 (4·3–21·4)**
**East Asia**	**20741·4 (18573·6–23593·4)**	**445·8 (359·0–559·5)**	**2·1 (1·8–2·6)**	**26798·8 (23208·3–31180·8)**	**657·3 (510·6–841·6)**	**2·5 (2·0–3·0)**	**14·3 (3·2–25·0)**
China	20156·6 (18075·4–22898·2)	432·6 (350·2–539·6)	2·1 (1·8–2·6)	25999·8 (22535·0–30187·2)	637·3 (495·5–814·7)	2·5 (2·0–3·0)	14·3 (3·2–25·1)
Democratic People's Republic of Korea	321·5 (269·5–382·7)	6·7 (4·5–10·2)	2·1 (1·5–3·0)	420·0 (311·4–552·5)	9·5 (5·8–14·5)	2·3 (1·6–3·2)	8·8 (0·2–15·8)
Taiwan, China	263·2 (224·0–311·3)	6·5 (4·4–9·5)	2·5 (1·7–3·5)	379·0 (282·0–490·1)	10·6 (6·5–17·5)	2·8 (2·0–3·9)	13·9 (0·2–27·9)
**Oceania**	**118·1 (97·1–147·5)**	**1·9 (1·2–3·0)**	**1·6 (1·0–2·5)**	**223·1 (170·8–288·2)**	**3·9 (2·3–6·2)**	**1·7 (1·1–2·6)**	**7·5 (1·1–13·7)**
American Samoa	0·6 (0·5–0·8)	0·0 (0·0–0·0)	1·8 (1·1–2·6)	0·7 (0·5–1·0)	0·0 (0·0–0·0)	1·9 (1·3–2·7)	6·3 (-6·4–18·1)
Cook Islands	0·3 (0·3–0·4)	0·0 (0·0–0·0)	2·1 (1·4–3·0)	0·4 (0·3–0·5)	0·0 (0·0–0·0)	2·3 (1·6–3·3)	11·4 (0·0–22·8)
Fiji	12·0 (9·9–14·3)	0·2 (0·1–0·3)	1·7 (1·1–2·5)	17·4 (13·2–23·6)	0·3 (0·2–0·5)	1·8 (1·2–2·6)	8·2 (0·1–17·5)
Guam	2·6 (2·2–3·1)	0·1 (0·0–0·1)	2·1 (1·4–3·1)	3·8 (2·8–4·9)	0·1 (0·0–0·1)	2·1 (1·5–3·0)	2·4 (-6·7–14·7)
Kiribati	1·2 (1·0–1·5)	0·0 (0·0–0·0)	1·5 (1·0–2·3)	2·0 (1·5–2·7)	0·0 (0·0–0·1)	1·6 (1·1–2·4)	5·6 (-1·2–13·2)
Marshall Islands	0·7 (0·6–0·9)	0·0 (0·0–0·0)	1·5 (0·9–2·2)	0·9 (0·7–1·3)	0·0 (0·0–0·0)	1·7 (1·1–2·5)	17·9 (6·2–30·1)
Micronesia (Federated States of)	1·5 (1·2–1·8)	0·0 (0·0–0·0)	1·5 (1·0–2·3)	1·8 (1·3–2·3)	0·0 (0·0–0·0)	1·7 (1·1–2·5)	11·9 (2·8–21·5)
Nauru	0·2 (0·1–0·2)	0·0 (0·0–0·0)	1·6 (1·0–2·4)	0·2 (0·1–0·3)	0·0 (0·0–0·0)	1·7 (1·1–2·5)	5·6 (0·7–10·8)
Niue	0·0 (0·0–0·0)	0·0 (0·0–0·0)	1·9 (1·3–2·7)	0·0 (0·0–0·0)	0·0 (0·0–0·0)	1·9 (1·4–2·8)	3·2 (-1·8–9·1)
Northern Mariana Islands	1·0 (0·8–1·2)	0·0 (0·0–0·0)	2·1 (1·3–3·2)	0·9 (0·7–1·3)	0·0 (0·0–0·0)	2·1 (1·4–2·9)	-0·8 (-22·5–20·6)
Palau	0·4 (0·3–0·5)	0·0 (0·0–0·0)	1·9 (1·3–2·9)	0·4 (0·3–0·6)	0·0 (0·0–0·0)	2·0 (1·4–3·0)	5·8 (-11·5–22·4)
Papua New Guinea	84·9 (69·1–107·2)	1·3 (0·8–2·1)	1·6 (1·0–2·4)	173·1 (127·1–231·4)	3·0 (1·7–4·9)	1·7 (1·1–2·6)	8·3 (1·3–16·2)
Samoa	2·3 (1·9–2·8)	0·0 (0·0–0·1)	1·7 (1·1–2·5)	3·1 (2·3–4·2)	0·1 (0·0–0·1)	1·8 (1·2–2·6)	4·5 (-1·6–11·3)
Solomon Islands	6·2 (5·1–7·7)	0·1 (0·1–0·1)	1·5 (1·0–2·3)	11·3 (8·2–15·2)	0·2 (0·1–0·3)	1·7 (1·1–2·5)	8·1 (-0·2–18·1)
Tokelau	0·0 (0·0–0·0)	0·0 (0·0–0·0)	1·8 (1·2–2·6)	0·0 (0·0–0·0)	0·0 (0·0–0·0)	1·9 (1·3–2·7)	5·3 (-0·8–11·7)

Tonga	1·2 (1·0–1·4)	0·0 (0·0–0·0)	1·7 (1·1–2·4)	1·4 (1·0–2·0)	0·0 (0·0–0·0)	1·8 (1·2–2·7)	8·3 (1·7–15·4)
Tuvalu	0·2 (0·1–0·2)	0·0 (0·0–0·0)	1·7 (1·2–2·5)	0·2 (0·2–0·3)	0·0 (0·0–0·0)	1·8 (1·2–2·6)	2·2 (-3·5–9·5)
Vanuatu	2·7 (2·3–3·4)	0·0 (0·0–0·1)	1·5 (1·0–2·3)	5·2 (3·9–7·0)	0·1 (0·0–0·1)	1·6 (1·1–2·4)	7·4 (0·3–14·9)
**Southeast Asia**	**6546·6 (5634·8–7794·1)**	**114·9 (81·8–160·6)**	**1·8 (1·3–2·4)**	**11925·8 (10029·4–14420·5)**	**239·6 (176·6–343·2)**	**2·0 (1·6–2·7)**	**14·7 (7·3–21·1)**
Cambodia	193·8 (160·1–239·3)	2·9 (1·8–4·5)	1·5 (0·9–2·2)	341·8 (256·2–447·3)	6·1 (3·6–9·6)	1·8 (1·2–2·6)	21·8 (12·8–31·0)
Indonesia	2155·7 (1809·8–2586·4)	35·2 (27·0–46·0)	1·6 (1·3–2·0)	4267·7 (3518·6–5156·5)	81·1 (61·2–105·6)	1·9 (1·5–2·4)	16·4 (12·4–20·4)
Lao People's Democratic Republic	63·9 (52·6–79·2)	0·9 (0·5–1·4)	1·4 (0·9–2·1)	120·7 (87·0–159·1)	2·0 (1·1–3·4)	1·6 (1·0–2·5)	20·0 (12·3–27·0)
Malaysia	364·6 (302·8–440·6)	6·5 (4·3–9·9)	1·8 (1·2–2·6)	777·6 (584·9–1036·1)	16·3 (10·0–27·0)	2·1 (1·5–3·1)	17·8 (9·8–26·5)
Maldives	5·1 (4·2–6·0)	0·1 (0·1–0·1)	1·6 (1·1–2·5)	11·1 (8·3–14·6)	0·3 (0·2–0·4)	2·4 (1·6–3·5)	44·3 (28·6–63·9)
Mauritius	39·4 (34·3–45·9)	0·8 (0·5–1·3)	2·1 (1·4–3·1)	46·3 (34·8–59·7)	1·0 (0·7–1·7)	2·2 (1·6–3·2)	8·3 (-1·6–21·0)
Myanmar	351·6 (291·4–431·6)	5·1 (3·2–8·1)	1·5 (1·0–2·2)	709·6 (516·3–970·0)	12·3 (7·6–19·3)	1·7 (1·2–2·5)	19·0 (11·2–26·2)
Philippines	1095·5 (920·5–1305·7)	19·0 (14·6–24·6)	1·7 (1·4–2·2)	2279·4 (1900·7–2757·3)	41·7 (31·4–53·7)	1·8 (1·5–2·3)	5·6 (3·9–7·3)
Seychelles	1·1 (1·0–1·4)	0·0 (0·0–0·0)	1·8 (1·2–2·7)	2·0 (1·5–2·6)	0·0 (0·0–0·1)	2·1 (1·4–3·0)	15·5 (6·3–26·2)
Sri Lanka	339·2 (292·9–394·7)	6·3 (4·0–9·5)	1·8 (1·2–2·7)	468·2 (357·6–610·5)	10·3 (6·3–16·5)	2·2 (1·5–3·1)	19·2 (8·7–29·2)
Thailand	1030·9 (865·6–1239·5)	20·9 (13·5–32·3)	2·0 (1·3–2·9)	1455·4 (1077·0–1893·1)	35·5 (22·3–55·4)	2·4 (1·7–3·5)	21·2 (0·1–43·2)
Timor-Leste	11·6 (9·7–14·2)	0·2 (0·1–0·3)	1·5 (1·0–2·2)	22·5 (16·4–30·2)	0·4 (0·2–0·6)	1·6 (1·0–2·3)	4·2 (-3·5–11·2)
Viet Nam	894·1 (769·7–1064·1)	17·2 (11·0–26·1)	1·9 (1·3–2·8)	1423·6 (1043·9–1866·5)	32·8 (19·3–53·6)	2·3 (1·6–3·4)	20·1 (8·0–32·1)
**Sub-Saharan Africa**	**18773·9 (16249·2–22222·3)**	**343·8 (252·0–478·7)**	**1·8 (1·4–2·4)**	**36729·8 (30976·5–44026·4)**	**720·8 (516·6–1014·7)**	**2·0 (1·5–2·6)**	**7·1 (5·1–9·4)**
**Central Sub-Saharan Africa**	**2512·5 (2067·1–3100·6)**	**45·8 (28·9–73·0)**	**1·8 (1·2–2·7)**	**5294·8 (4132·9–6875·3)**	**102·7 (62·7–165·3)**	**1·9 (1·2–2·9)**	**6·3 (0·2–10·8)**
Angola	530·3 (432·3–662·5)	9·7 (6·0–15·3)	1·8 (1·1–2·8)	1295·3 (963·8–1718·1)	25·3 (14·7–40·3)	2·0 (1·2–2·9)	7·1 (-0·2–14·3)
Central African Republic	137·1 (111·5–169·1)	2·4 (1·5–3·9)	1·7 (1·1–2·7)	231·1 (168·0–310·0)	4·2 (2·5–6·8)	1·8 (1·1–2·8)	4·2 (-1·7–10·6)
Congo	129·3 (111·0–154·1)	2·4 (1·5–3·7)	1·8 (1·2–2·8)	245·6 (178·5–330·5)	5·2 (3·0–8·5)	2·1 (1·4–3·1)	14·2 (3·2–24·5)
Democratic Republic of the Congo	1645·6 (1353·8–2038·0)	30·0 (18·9–48·2)	1·8 (1·2–2·7)	3372·3 (2487·1–4501·4)	64·9 (38·8–106·1)	1·9 (1·2–2·9)	5·3 (-1·9–11·7)
Equatorial Guinea	22·5 (18·4–27·3)	0·4 (0·3–0·6)	1·8 (1·2–2·8)	64·5 (48·8–87·8)	1·3 (0·8–2·2)	2·1 (1·3–3·1)	12·8 (5·6–20·4)
Gabon	47·8 (40·1–57·5)	0·9 (0·6–1·4)	2·0 (1·3–2·8)	85·9 (65·0–110·6)	1·8 (1·1–2·9)	2·1 (1·4–3·1)	8·3 (2·8–15·5)
**Eastern Sub-Saharan Africa**	**9303·7 (8045·4–10964·0)**	**167·3 (124·7–226·8)**	**1·8 (1·4–2·4)**	**18345·4 (15430·4–21979·9)**	**357·7 (261·2–493·9)**	**2·0 (1·5–2·6)**	**8·4 (6·0–11·3)**
Burundi	168·7 (140·5–204·2)	3·1 (2·0–4·8)	1·8 (1·2–2·6)	369·3 (265·5–498·7)	7·0 (4·1–11·5)	1·9 (1·3–2·7)	2·9 (-4·1–10·7)
Comoros	12·1 (10·1–14·6)	0·2 (0·2–0·4)	1·9 (1·3–2·8)	22·7 (16·9–30·5)	0·5 (0·3–0·8)	2·1 (1·5–3·0)	10·6 (2·9–19·7)

Djibouti	14·6 (12·0–17·9)	0·3 (0·2–0·4)	1·9 (1·2–2·7)	39·2 (29·6–51·7)	0·8 (0·5–1·3)	2·1 (1·4–3·1)	14·5 (6·8–23·4)
Eritrea	96·2 (81·1–118·4)	1·8 (1·1–2·7)	1·8 (1·2–2·7)	200·7 (148·7–264·3)	3·9 (2·4–6·2)	2·0 (1·3–2·9)	8·3 (0·2–14·7)
Ethiopia	4555·7 (3850·5–5416·2)	80·0 (61·5–103·1)	1·8 (1·4–2·2)	8642·0 (7127·9–10533·0)	168·3 (125·7–220·8)	1·9 (1·6–2·4)	10·9 (6·8–15·2)
Kenya	801·0 (697·5–944·4)	15·7 (12·3–20·1)	2·0 (1·6–2·4)	1533·4 (1330·9–1793·8)	31·7 (24·7–40·1)	2·1 (1·7–2·5)	5·4 (3·0–7·7)
Madagascar	369·9 (306·0–451·5)	6·8 (4·4–10·9)	1·8 (1·3–2·7)	844·7 (619·7–1142·5)	16·2 (9·6–26·3)	1·9 (1·3–2·8)	3·9 (-1·9–10·2)
Malawi	217·7 (186·6–257·1)	4·0 (2·6–6·0)	1·8 (1·2–2·7)	472·9 (346·2–626·6)	8·8 (5·5–14·9)	1·9 (1·3–2·7)	1·3 (-5·3–8·9)
Mozambique	425·1 (355·3–514·3)	7·9 (5·2–12·3)	1·9 (1·3–2·8)	925·2 (677·3–1242·0)	17·1 (10·3–28·4)	1·8 (1·2–2·7)	-1·2 (-8·9–5·8)
Rwanda	236·2 (197·4–286·1)	4·3 (2·8–6·6)	1·8 (1·2–2·7)	456·5 (338·3–593·8)	9·5 (5·8–15·1)	2·1 (1·4–3·0)	15·3 (8·6–23·2)
Somalia	233·0 (191·3–283·1)	4·1 (2·6–6·4)	1·8 (1·2–2·6)	615·4 (455·3–821·2)	10·8 (6·3–18·0)	1·7 (1·1–2·6)	-0·8 (-8·1–7·4)
South Sudan	165·4 (136·0–203·8)	3·0 (1·9–4·6)	1·8 (1·2–2·7)	256·5 (189·3–347·6)	4·8 (2·8–8·2)	1·9 (1·3–2·7)	3·0 (-6·0–11·8)
Uganda	1021·4 (896·7–1166·3)	17·5 (11·6–25·6)	1·7 (1·1–2·5)	1840·7 (1370·6–2452·5)	35·9 (21·8–58·4)	2·0 (1·3–2·8)	13·9 (5·8–23·7)
United Republic of Tanzania	799·9 (668·0–976·2)	15·3 (10·1–23·1)	1·9 (1·3–2·7)	1636·7 (1219·7–2200·1)	32·9 (19·9–54·4)	2·0 (1·4–2·9)	5·3 (-1·4–12·2)
Zambia	186·6 (160·8–217·9)	3·2 (2·1–4·9)	1·7 (1·2–2·5)	489·4 (363·5–660·6)	9·4 (5·7–15·5)	1·9 (1·3–2·9)	11·3 (3·5–19·5)
**Southern Sub-Saharan Africa**	**1479·7 (1301·4–1705·6)**	**36·6 (28·3–47·3)**	**2·5 (2·0–3·1)**	**2915·0 (2497·3–3437·1)**	**75·6 (57·0–101·6)**	**2·6 (2·1–3·3)**	**4·9 (2·1–7·7)**
Botswana	42·0 (35·0–50·8)	1·0 (0·6–1·4)	2·3 (1·5–3·4)	89·8 (66·9–122·2)	2·3 (1·4–3·8)	2·6 (1·7–3·9)	14·1 (5·4–22·4)
Eswatini	22·8 (19·5–26·9)	0·5 (0·3–0·7)	2·1 (1·4–3·2)	47·2 (34·1–63·0)	1·1 (0·6–1·7)	2·2 (1·5–3·3)	4·4 (-2·7–11·9)
Lesotho	60·5 (50·2–72·5)	1·4 (0·9–2·2)	2·3 (1·6–3·4)	102·7 (77·8–135·9)	2·3 (1·4–3·7)	2·2 (1·5–3·2)	-4·8 (-13·4–6·6)
Namibia	34·1 (29·4–39·6)	0·7 (0·5–1·1)	2·2 (1·5–3·1)	73·6 (54·1–98·2)	1·7 (1·1–2·8)	2·4 (1·6–3·5)	8·1 (-0·5–16·8)
South Africa	1155·2 (1020·8–1329·8)	29·3 (23·2–37·3)	2·5 (2·1–3·1)	2303·8 (1972·7–2715·5)	61·5 (47·5–79·7)	2·7 (2·2–3·3)	5·2 (2·1–8·3)
Zimbabwe	165·0 (139·0–200·9)	3·7 (2·5–5·3)	2·2 (1·6–3·2)	298·0 (219·4–387·4)	6·7 (4·1–10·6)	2·2 (1·5–3·3)	0·8 (-5·8–7·9)
**Western Sub-Saharan Africa**	**5478·1 (4685·7–6479·6)**	**94·1 (69·6–128·8)**	**1·7 (1·3–2·3)**	**10174·6 (8519·0–12346·2)**	**184·8 (131·3–264·2)**	**1·8 (1·4–2·5)**	**5·6 (2·8–8·7)**
Benin	138·2 (114·6–170·0)	2·4 (1·5–3·7)	1·7 (1·2–2·5)	324·3 (241·2–431·6)	5·9 (3·6–9·2)	1·8 (1·2–2·6)	5·7 (0·3–10·3)
Burkina Faso	243·0 (208·1–286·9)	4·2 (2·9–6·2)	1·7 (1·2–2·5)	514·6 (387·1–691·5)	9·2 (5·7–15·1)	1·8 (1·2–2·6)	2·6 (-3·2–8·2)
Cabo Verde	13·0 (11·0–15·6)	0·3 (0·2–0·4)	2·0 (1·4–2·8)	25·1 (18·9–33·4)	0·6 (0·4–0·9)	2·3 (1·6–3·2)	14·2 (7·1–22·0)
Cameroon	346·6 (292·0–417·4)	5·9 (3·8–9·0)	1·7 (1·2–2·5)	831·0 (619·1–1113·5)	15·3 (9·1–25·0)	1·8 (1·3–2·7)	8·1 (2·8–14·8)
Chad	221·2 (190·0–257·6)	3·7 (2·5–5·5)	1·7 (1·2–2·4)	449·8 (327·8–596·8)	7·5 (4·5–11·8)	1·7 (1·1–2·4)	0·3 (-6·8–7·8)
Côte d'Ivoire	310·2 (265·4–373·4)	5·2 (3·4–8·0)	1·7 (1·1–2·4)	565·1 (427·0–755·3)	10·6 (6·5–17·6)	1·9 (1·3–2·7)	11·4 (4·3–19·0)
Gambia	43·4 (36·2–52·4)	0·8 (0·5–1·1)	1·7 (1·2–2·6)	86·0 (63·5–112·5)	1·5 (0·9–2·4)	1·8 (1·2–2·6)	1·5 (-5·6–8·6)

Ghana	444·6 (381·0–526·1)	8·1 (5·4–12·1)	1·8 (1·3–2·7)	954·9 (705·9–1273·6)	18·3 (11·2–29·5)	1·9 (1·3–2·8)	4·4 (-0·9–10·4)
Guinea	176·1 (147·0–213·6)	3·0 (2·0–4·6)	1·7 (1·2–2·4)	329·6 (247·0–439·9)	5·7 (3·5–9·1)	1·7 (1·2–2·5)	2·3 (-6·3–11·0)
Guinea-Bissau	26·0 (21·2–32·3)	0·4 (0·3–0·6)	1·6 (1·1–2·4)	51·6 (38·4–70·0)	0·9 (0·5–1·4)	1·7 (1·1–2·5)	7·8 (2·6–13·9)
Liberia	67·2 (56·0–83·3)	1·1 (0·8–1·7)	1·7 (1·2–2·4)	145·7 (109·5–195·8)	2·7 (1·6–4·6)	1·8 (1·2–2·7)	9·1 (2·1–17·4)
Mali	167·3 (140·5–202·6)	2·8 (1·9–4·3)	1·7 (1·2–2·5)	393·8 (287·2–532·6)	6·7 (3·9–10·8)	1·7 (1·2–2·5)	0·2 (-7·4–6·4)
Mauritania	44·7 (38·2–53·9)	0·8 (0·6–1·2)	1·8 (1·3–2·6)	88·0 (66·1–116·7)	1·7 (1·1–2·6)	1·9 (1·4–2·8)	4·9 (-0·7–10·3)
Niger	207·5 (171·4–254·7)	3·4 (2·2–5·4)	1·7 (1·1–2·4)	452·9 (334·1–588·8)	7·6 (4·7–11·9)	1·7 (1·2–2·5)	1·6 (-4·3–8·2)
Nigeria	2635·5 (2236·1–3121·3)	45·1 (35·1–57·2)	1·7 (1·4–2·1)	4127·8 (3473·7–4898·0)	75·2 (58·2–96·4)	1·8 (1·5–2·3)	6·6 (4·8–8·5)
Sao Tome and Principe	2·5 (2·1–3·1)	0·0 (0·0–0·1)	1·7 (1·2–2·4)	4·8 (3·6–6·5)	0·1 (0·1–0·2)	1·9 (1·3–2·8)	14·9 (6·8–25·2)
Senegal	185·5 (159·8–218·6)	3·2 (2·2–4·7)	1·7 (1·2–2·5)	394·7 (295·6–521·4)	7·2 (4·5–11·5)	1·8 (1·3–2·7)	5·2 (-0·6–10·7)
Sierra Leone	100·0 (83·6–121·9)	1·8 (1·2–2·7)	1·8 (1·2–2·5)	213·0 (155·1–285·4)	3·9 (2·4–6·2)	1·8 (1·2–2·7)	3·7 (-4·1–11·1)
Togo	105·7 (88·4–130·1)	1·8 (1·2–2·8)	1·7 (1·2–2·5)	222·0 (163·9–291·5)	4·2 (2·5–6·7)	1·9 (1·3–2·7)	9·5 (2·1–17·7)

Note: UI = Uncertainty interval

Table S7: Sensitivity analysis to assess the impact of indirect effects on the bias correction model

Covariate	Coefficient (95% UI)	
	All data	Excluding indirect data
Estimate represents any use of antidepressants	0.179 (0.072 to 0.287)	0.123 (-0.015 to 0.260)
Estimate represents any mental health service utilisation	0.062 (-0.167 to 0.292)	0.084 (-0.171 to 0.338)
Interaction between estimate represents any mental health service utilisation and HAQI*	-0.012 (-0.024 to -0.001)	-0.011 (-0.024 to 0.001)
